# Mouse models of 17q21.31 microdeletion and microduplication syndromes highlight the importance of *Kansl1* for cognition

**DOI:** 10.1371/journal.pgen.1006886

**Published:** 2017-07-13

**Authors:** Thomas Arbogast, Giovanni Iacono, Claire Chevalier, Nurudeen O. Afinowi, Xander Houbaert, Matthijs C. van Eede, Christine Laliberte, Marie-Christine Birling, Katrin Linda, Hamid Meziane, Mohammed Selloum, Tania Sorg, Nael Nadif Kasri, David A. Koolen, Henk G. Stunnenberg, R. Mark Henkelman, Maksym Kopanitsa, Yann Humeau, Bert B. A. De Vries, Yann Herault

**Affiliations:** 1 Institut de Génétique et de Biologie Moléculaire et Cellulaire, Université de Strasbourg, Illkirch, France; 2 Centre National de la Recherche Scientifique, UMR7104, Illkirch, France; 3 Institut National de la Santé et de la Recherche Médicale, U964, Illkirch, France; 4 Université de Strasbourg, Illkirch, France; 5 Department of Molecular Biology, Radboud Institute for Molecular Life Sciences, Radboud University, Nijmegen, The Netherlands; 6 Synome Ltd, Moneta Building, Babraham Research Campus, Cambridge, United Kingdom; 7 Interdisciplinary Institute for Neuroscience, CNRS, UMR5297, Bordeaux University, Bordeaux, France; 8 Mouse Imaging Center (MICe), The Hospital for Sick Children Toronto, Toronto, Ontario, Canada; 9 CELPHEDIA, PHENOMIN, Institut Clinique de la Souris (ICS), CNRS, INSERM, University of Strasbourg, Illkirch-Graffenstaden, France; 10 Department of Human Genetics, Donders Institute for Brain, Cognition and Behaviour, Nijmegen, the Netherlands; University of Pennsylvania, UNITED STATES

## Abstract

Koolen-de Vries syndrome (KdVS) is a multi-system disorder characterized by intellectual disability, friendly behavior, and congenital malformations. The syndrome is caused either by microdeletions in the 17q21.31 chromosomal region or by variants in the *KANSL1* gene. The reciprocal 17q21.31 microduplication syndrome is associated with psychomotor delay, and reduced social interaction. To investigate the pathophysiology of 17q21.31 microdeletion and microduplication syndromes, we generated three mouse models: 1) the deletion (*Del/+*); or 2) the reciprocal duplication (*Dup/+*) of the 17q21.31 syntenic region; and 3) a heterozygous *Kansl1* (*Kans1*^*+/-*^) model. We found altered weight, general activity, social behaviors, object recognition, and fear conditioning memory associated with craniofacial and brain structural changes observed in both *Del/+* and *Dup/+* animals. By investigating hippocampus function, we showed synaptic transmission defects in *Del/+* and *Dup/+* mice. Mutant mice with a heterozygous loss-of-function mutation in *Kansl1* displayed similar behavioral and anatomical phenotypes compared to *Del/+* mice with the exception of sociability phenotypes. Genes controlling chromatin organization, synaptic transmission and neurogenesis were upregulated in the hippocampus of *Del/+* and *Kansl1*^*+/-*^ animals. Our results demonstrate the implication of *KANSL1* in the manifestation of KdVS phenotypes and extend substantially our knowledge about biological processes affected by these mutations. Clear differences in social behavior and gene expression profiles between *Del/+* and *Kansl1*^*+/-*^ mice suggested potential roles of other genes affected by the 17q21.31 deletion. Together, these novel mouse models provide new genetic tools valuable for the development of therapeutic approaches.

## Author summary

The 17q21.31 deletion syndrome, also named Koolen-de Vries syndrome (KdVS), is a rare copy number variants associated in humans with intellectual disability, friendly behavior, congenital malformations. The syndrome is caused either by microdeletions in the 17q21.31 region or by variants in the *KANSL1* gene in human. The reciprocal 17q21.31 microduplication syndrome is not so well characterized. To investigate the pathophysiology of the syndromes, we studied the deletion, the duplication of the syntenic region and a heterozygous *Kansl1* mutant in the mouse. We found affected morphology and cognition, similar to human condition, with genes controlling chromatin organization, synaptic transmission and neurogenesis dysregulated in the hippocampus of *KdVS* models. In addition we found that synaptic transmission was altered in KdVS mice. Our results demonstrate the implication of *KANSL1* in the manifestation of KdVS and extend substantially our knowledge about altered biological processes. Nevertheless, phenotypic differences between deletion and *Kansl1*^*+/-*^ models suggested roles of other genes affected by the 17q21.31 deletion.

## Introduction

The Koolen-de Vries syndrome (KdVS) has a prevalence estimated at 1/55,000 based upon CNV studies[1–3] and was primary described as a consequence of the 17q21.31 microdeletion. Patients with KdVS present characteristic facial dysmorphisms[4] and clinical features including intellectual disability, friendly behavior, hypotonia, and several brain anomalies[5–7]. Microdeletions and microduplications of genomic fragments in the 17q21.3 region ranging from 400 to 800kb have been found in individuals with intellectual disability[6, 8]; these genomic fragments include five protein-coding genes: *CRHR1*, *SPPL2C*, *MAPT*, *STH*, and *KANSL1*. The reciprocal duplication is much more rare than the deletion. To our knowledge, only eight patients have been described in the literature[8–12]. The symptoms are heterogeneous and include craniofacial malformations, microcephaly, psychomotor delay, poor verbal and motor skills, and reduced social interaction[8]. Two cases out of eight have been diagnosed with autism spectrum disorder (ASD).

Loss-of-function mutations and atypical deletions restricted to the *KANSL1* gene, encoding the KAT8 Regulatory NSL Complex Subunit 1, have been found in several KdVS patients. Interestingly, phenotypic comparison of both the 17q21.31 microdeletion and *KANSL1* heterozygous mutation patients show similar clinical severity, implicating that haploinsufficiency of *KANSL1* is sufficient to cause the full manifestation of KdVS phenotype[3, 13–15]. KANSL1 is a member of the evolutionarily conserved nonspecific lethal (NSL) complex that controls various cellular functions, including transcription regulation and stem cell identity maintenance and reprogramming[16, 17]. The NSL complex contains the histone acetyltransferase MOF (males absent on the first) encoded by *KAT8* which acetylates histone H4 on lysine 16 (H4K16) and with lower efficiency on lysines 5 and 8 (H4K5 and H4K8, respectively) to facilitate transcriptional activation[18, 19]. Recent studies in flies have shown that KANSL1 acts as a scaffold protein interacting with four NSL subunits including WDR5 which plays a critical role in assembling distinct histone-modifying complexes with different epigenetic regulatory functions[20].

Genes within the human 17q21.31 region are highly conserved on mouse chromosome 11E1. *Crhr1*, *Sppl2c*, *Mapt* and *Kansl1* orthologs have all been found in the same orientation as in the human H1 haplotype. To investigate the pathophysiology of KdVS and microduplication syndrome, we generated first a mutant mice bearing deletion (*Del/+*), and duplication (*Dup/+*) of the 17q21.31-homologous *Arf2-Kansl1* genetic interval and looked for phenotypes related to the human condition. We studied behavior, cognition, craniofacial and brain morphology of single Deletion carried compared to wild type and pseudo-disomic (Del/Dup) controls. Then we compared these data to results obtained with mutant mice for *Kansl1* and extended our analysis to gene expression. We found a large phenotypic overlap with altered molecular mechanisms controlling the hippocampus synaptic response.

## Results

### Deletion of the *Arf2*–*Kansl1* genetic interval impairs viability and alters post-natal development with reduced size and body weight

**Fig 1:**
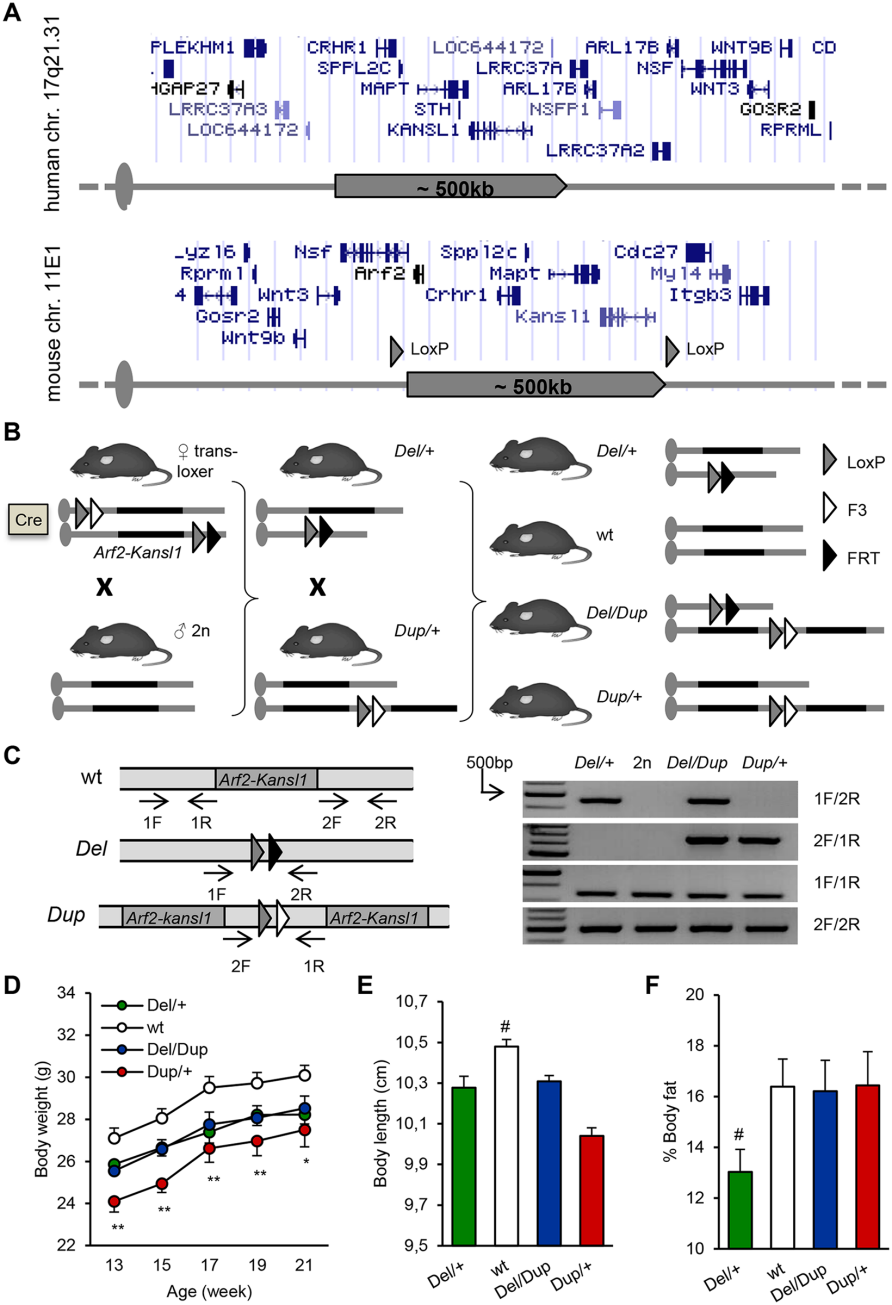
Mouse models of 17q21.31 rearrangements. **(A)** Top: haplotype H1 of the human 17q21.31 region. All genomic positions are given according to the UCSC human genome browser GRCh38/hg38. Bottom: 17q21.31 syntenic region on mouse chromosome 11E1. All genomic positions are given according to UCSC mouse genome browser GRCm38/mm10. **(B)** Strategy for *in vivo* Cre-mediated recombination and targeted meiotic recombination (TAMERE) crossing strategy. LoxP sites were inserted upstream of *Arf2* and downstream of *Kansl1*. The breeding strategy aimed to have trans-loxer females expressing the *Hprt*^*tm1(cre)Mnn*^ transgene and carrying the two loxP sites in a *trans* configuration. The last step consisted of mating trans-loxer females with wt males to generate progeny carrying the deletion (*Del/+*) or the duplication (*Dup/+*) of the *Arf2-Kansl1* region. *Del/+* and *Dup/+* animals were crossed together to generate *Del-Dup* cohorts. **(C)** Molecular validation. PCR products specific for the *Del* and *Dup* alleles are 448-bp and 653-bp respectively. **(D)** Evolution of body weight (g) of adult animals (F_(3,300)_ = 5.529, *P* = 0.002; *Del/+* vs wt: P = 0.101, *Dup/+* vs wt: *P* = 0.002, *Del/Dup* vs wt: P = 0.109). **(E)** Body length (distance from the snout to the tail base) of 20-week old animals (H_(3, 55)_ = 28.036, *P* < 0.001; *Del/+* vs wt: *P* = 0.004, *Dup/+* vs wt: *P* < 0.001, *Del/Dup* vs wt: *P* = 0.003). **(F)** Body fat percentage of 20-week-old animals measured by qNMR (H_(3, 55)_ = 8.120, *P* = 0.044; *Del/+* vs wt: *P* = 0.027). Data are represented as the mean ± s.e.m. Cohort used included 18 *Del/+*, 24 wt, 11 *Del/Dup*, and 11 *Dup/+* animals. **(A)** Repeated Measures ANOVA “genotype” analysis, Tukey's *post hoc* test. **(B, C, E, F)** Kruskal-Wallis analysis, Mann-Whitney *U* test. *P < 0.05 vs wt, **P < 0.01 vs wt, ***P < 0.001 vs wt, ^#^ *P* < 0.05 vs all other groups.

*Del/+* and *Dup/+* mice were generated on the C57BL/6N (B6N) genetic background (see supplementary information; Fig 1). In comparison with wild-type (wt) littermates, he *Del* allele frequency was reduced significantly while the transmission of the *Dup* allele and the *Del/Dup* carriers were not affected (Table 1), thus demonstrating that lethality is associated with the deletion on the B6N background.

**Table 1:** Transmission rates of the *Arf2-Kansl1* deletion (*Del*) and duplication (*Dup*) alleles recorded at weaning. A reduced transmission rate of the *Del* allele was observed. Combination of both *Del* and *Dup* alleles led to the rescue of the *Del* allele-associated lethality.

Crossing	Genotype	Observed number	Observed ratio	χ^2^	*p*
*Del/+* × wt	wt	102	63.4%	5.74	0.02
	*Del/+*	59	36.6%		
*Dup/+* × wt	2n	186	55.0%	1.71	0.20
	*Dup/+*	152	45.0%		
*Del/+* × *Dup/+*	wt	71	37.6%	11.9	5.5x10^−04^
	*Del/+*	14	7.4%	23.4	1.3x10^−06^
	*Dup/+*	48	25.4%	0.01	0.91
	*Del/Dup*	56	29.6%	1.62	0.20

We generated and characterized a compound *Del-Dup* cohort with littermates carrying four genotypes: *Del/+*, wt, *Del/Dup*, and *Dup/+*. First, we followed general parameters. Compared to wt mice, *Dup/+* mice were underweight, whereas *Del/+* and *Del/Dup* mice were not (Fig 1d). At 20 weeks of age. *Del/+*, *Dup/+*, and *Del/Dup* animals showed significantly reduced body length compared to wt littermates (Fig 1e). In comparison with wt, *Del/+* littermates showed lower adiposity levels (Fig 1f) at the same age. Nevertheless, we did not detect any notable differences in feeding behavior between mutant and wt animals during a circadian activity test (S1 Fig).

### Deletion of the *Arf2*–*Kansl1* region decreases circadian activity and alters recognition and associative memory

**Fig 2:**
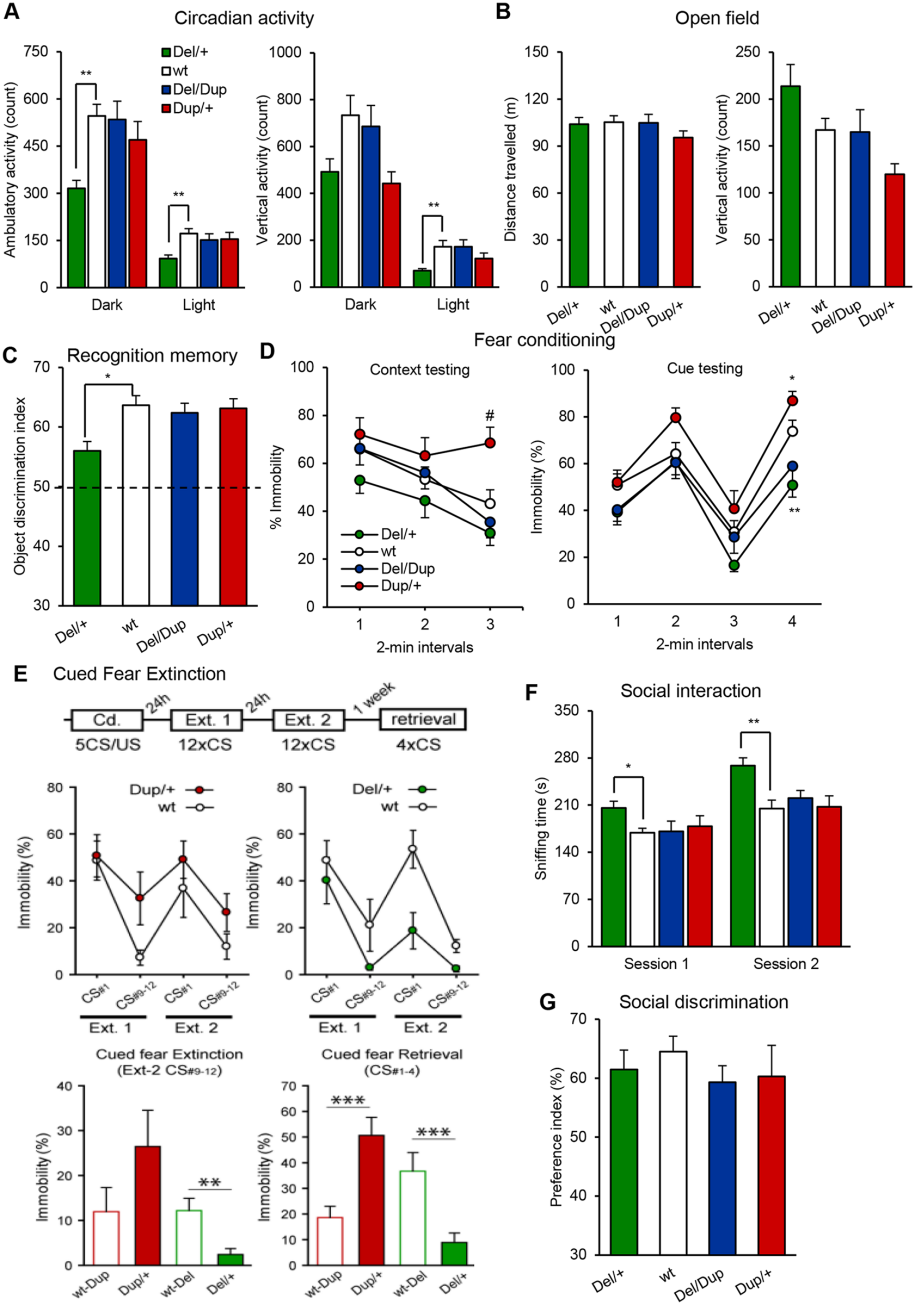
Behavioral characterization of *Del-Dup* cohorts. **(A)** Circadian activity test. Graphs plot the spontaneous ambulatory activity (count) and the vertical activity/rears (count) during dark and light phases. Del/+ mice showed reduced ambulatory activity during the dark phase (F_(3,46)_ = 5.791, *P* = 0.002; *Del/+* vs wt: *P* = 0.002) and the light phase (F_(3,46)_ = 4.260, *P* = 0.010; *Del/+* vs wt: *P* = 0.006) as well as reduced rearing behavior during the light phase (H_(3, 46)_ = 12.861, *P* = 0.005; *Del/+* vs wt: *P* = 0.002) **(B)** Open field test. Distance travelled (m), and vertical activity/rears (count) over 30 min of testing. **(C)** Novel object recognition test. Discrimination index was calculated as the ratio of time spent exploring the novel object vs the total time for object sniffing in the choice trial after a 3 h retention delay. The dashed line denotes a chance level of 50% (F_(3,43)_ = 3.081, *P* = 0.037; *Del/+* vs wt: *P* = 0.040). **(D)** Fear conditioning test. Plots represent the percentage of time spent freezing during test sessions. The 6-min context session was run 24 h after conditioning. The 8-min cue session was performed 5 h after the context session. A sequence of 2-min without cue and 2 min with light/auditory conditioning stimulus was repeated twice. **(E)** Cued fear extinction. Independent group of *Del /+* and *Dup/+* mutant mice with their respective wt control littermates were evaluated for cued fear extinction. Immobility was increased in *Dup/+* compared to control while it was decreased in the *Del/+* carriers during the two test periods for extinction (Ext1 (24 h) and Ext2 (48 h)). After one week the cued fear retrieval was observed in the *Dup/+* mice compared to their wt littermates whereas it was absent in the *Del/+* compared to their control. **(F-G)** Three-chamber sociability test. **(F)** Exploration time (s) of the first congener in the social interest session (session 1) and total exploration time of familiar and novel congeners in the social discrimination session (session 2). No delay was used between the two sessions. **(G)** Discrimination index was calculated as the ratio of time spent exploring the novel congener vs the familiar one. Data are represented as the mean ± s.e.m. Cohort used included 18 *Del/+*, 24 wt, 11 *Del/Dup*, and 11 *Dup/+* animals. *Post hoc* Tukey's and Mann Whitney U tests were performed following significant results in one-way ANOVA or Kruskal Wallis analysis, respectively. *P < 0.05 vs wt, **P < 0.01 vs wt, ***P < 0.001 vs wt, ^#^ *P* < 0.05 vs all other groups.

Patients with 17q21.31 CNVs have impaired intellectual and adaptive functioning[4, 5]. As a primary experiment, we looked at the activity and the *Del/+* and *Dup/+* mice displayed a normal circadian pattern (S1 Fig). However, in comparison with wt, *Del/+* mice showed reduced spontaneous locomotor activity (Fig 2a) as well as reduced rearing behavior during the light phase (Fig 2a). In the open field, no differences in exploration, locomotor activity, rearing behavior, or time spent in the center of the area were observed between mutant mice and wt littermates (Fig 2b; S1 Table; S2a–S2d Fig). In the elevated plus maze, *Del/+* and *Dup/+* mice explored the same number of arms and spent similar periods of time in open arms as those observed for wt (S1 Table). We evaluated motor coordination and learning using the rotarod test but no differences were observed between mutant *Del/+* and *Dup/+* mice, and wt mice (S2a–S2c Fig). Similarly, for the grip test, no differences in the muscular strength were observed between mutant mice and wt littermates (S2d Fig).

Spatial learning and memory was assessed in the Morris water maze, but similar acquisition and retention were observed in wt and mutant animals (S3 Fig). In the Y maze test, we found similar working memory parameters in wt and mutant animals (S2 Table). Next, we evaluated our models in the novel object recognition test. During the acquisition session, mice of all genotypes spent an equal amount of time exploring the sample object (S2 Table). After 3h of retention delay, *Del/+* mice are able to differentiate the novel versus the familiar object but show object discrimination deficits compared to wt whereas *Dup/+* and *Del/Dup* mice showed similar memory capacities (Fig 2c) compared to control.

Associative memory was evaluated with the fear conditioning test. No significant differences in the baseline and post-shock freezing levels were observed between mice of all genotypes (S2 Table). In the context session, *Dup/+* mice displayed a higher level of freezing with significant differences in the last 2 min of the test (F_(3,56)_ = 6.399, *P* < 0.001; *Dup/+* vs wt: *P* = 0.027; Fig 2d). The cued session was performed 5 h after the context session. During the presentation of the conditioning cue, all genotypes demonstrated higher freezing incidence (Fig 2d). During the second 2-min long cue, *Del/+* and *Dup/+* animals showed lower and higher levels of freezing, respectively, in comparison with wt littermates (H_(3, 56)_ = 20.609, *P* < 0.001; *Del/+* vs wt: *P* = 0.002, *Dup/+* vs wt: *P* = 0.036). To challenge cued fear extinction in those animals, we used another fear conditioning/extinction protocol with reinforced conditioned stimuli (CS) and long-term follow-up; we separated cohorts for the *Del/+* and for *Dup/+* with their own littermate controls. This revealed opposite effects on the capacity of *Del/+* and *Dup/+* animals to extinguish the fear response (Fig 2e). *Dup/+* animals presented with higher levels of freezing as compared to their wt littermates suggesting that the fear trace persists in those animals. In contrast, the freezing levels of *Del/+* animals were globally lower compared to those of their wt littermates, indicating that the fear memory trace is less stable in those animals.

### Deletion of the *Arf2*–*Kansl1* region increases sociability

To determine whether sociability traits were evident in our mouse models, we first used the three-chamber sociability test. Familiar and unfamiliar animals were of similar sex and genetic background than experimental animals but were of younger age in order to avoid aggressiveness. In the first phase of the test, the social interest session, *Del/+* mice spent relatively more time than wt littermates exploring the unfamiliar mouse (F_(3,51)_ = 3.447, *P* = 0.023; *Del/+* vs wt: *P* = 0.017; Fig 2f, Session 1; S4c Fig). In the second phase, the social discrimination session, *Del/+* mice interacted with familiar and novel congeners for longer periods compared to wt littermates as observed in the first session (F_(3,51)_ = 6.034, *P* = 0.001; *Del/+* vs wt: *P* = 0.002; Fig 2f, Session 2; S4d Fig). Nevertheless no differences in the percentage of time spent to explore the novel congener versus the familiar congener were observed between mutant mice and wt littermates (Fig 2e).

### Rearrangements of the *Arf2*–*Kansl1* region induce changes in craniofacial features and brain architecture

We studied the influence of 17q21.31-homologous CNVs on the mouse craniofacial structure. We analysed computed tomography (CT) cranial scans of animal heads combined with 3D reconstruction of skull images using 39 cranial landmarks (S5a Fig). Separate cohorts of *Del/+* and *Dup/+* females were used for the Euclidean distance matrix analysis[21] and MorphoJ analysis[22]. Skull size in *Del/+* (*T* = 0.115; S5b Fig) and *Dup/+* animals (*T* = 0.115; S5d Fig) was similar to that of wt littermates. The skull shape measurements were nominally altered in *Del/+* (*Z* = 0.077; S5c Fig) and *Dup/+* animals (*Z* = 0.052; S5e Fig) compared to those in wt littermates. Principal component analysis helped to identify change in the skull shape in *Del/+* and the *Dup/+* versus control littermates with the three main components (PC1 and PC2; S5f Fig) accounting for 59.2% of the total variance. Differences are more pronounced in the skull shape of the *Del/+* mice than in wt controls with a predominantly shorter nasal bone and a broadening of the face at the level of the zygomatic spine and squamosal junction.

**Fig 3:**
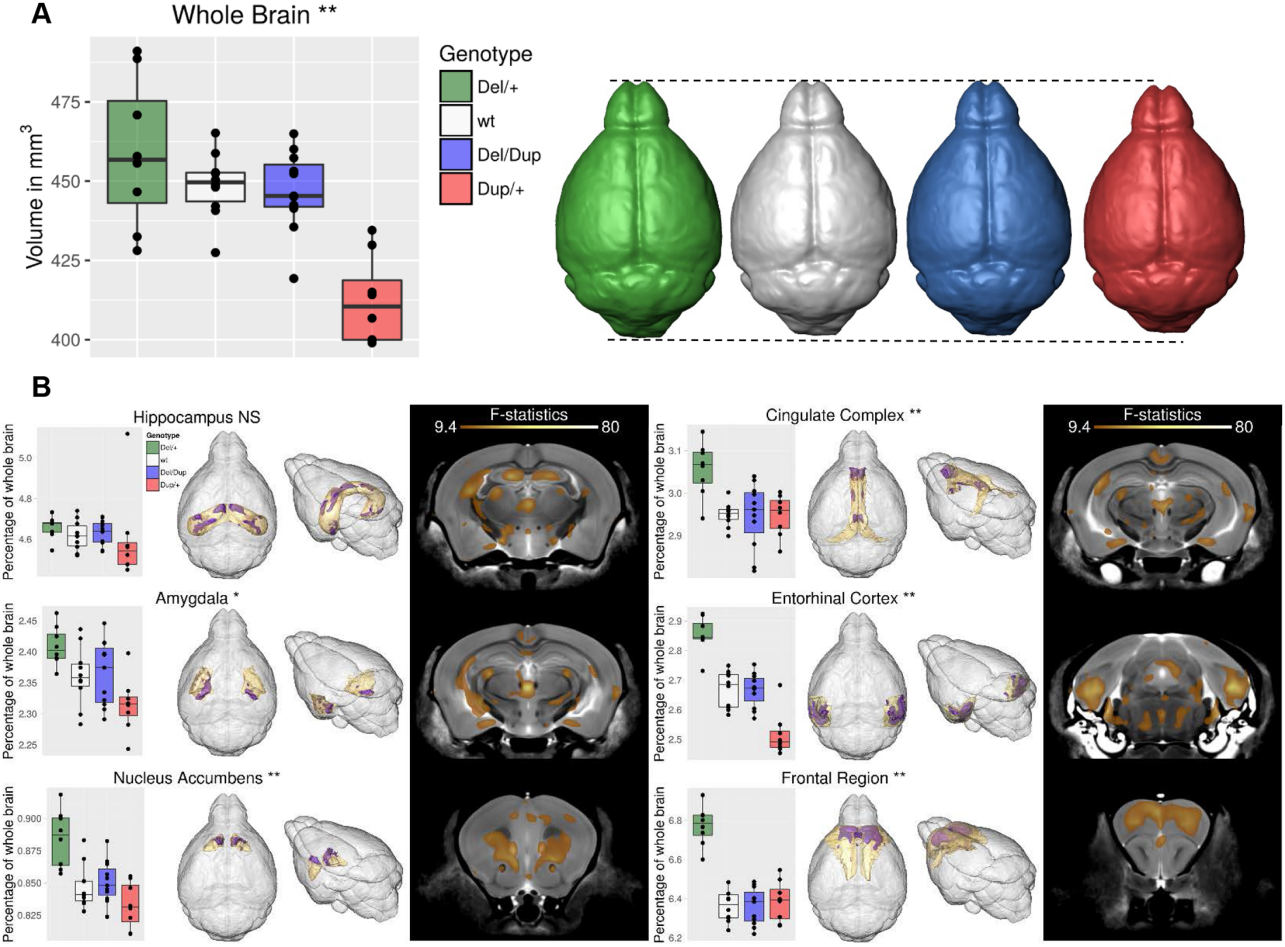
Differences in whole brain volume and relative volumes (normalized or overall brain volume) for the hippocampus, amygdala, nucleus accumbens, cingulate complex, entorhinal cortex and the frontal region of the 17q21.31 mouse models. All plots use box-and-whiskers diagrams. The box indicates data that lie within the 25th and 75th percentile. The horizontal line in the box specifies the median of the data, and the whiskers the full range of the data with the individual dots being the outliers. (**A**) Total brain volume is significantly different between the 4 genotypes at 0.1% FDR. (**B**) Relative volumes. For each structure, the following data are shown: on the left a bar graph indicating the relative size of the entire structure/region expressed as a percentage of total brain volume. Significance is based on the f-statistic resulting from comparing the 4 genotypes. NS = not significant, * = significant at 1% FDR, ** = significant at 0.1% FDR. In the center, the 3D surface renderings focus on significant changes for the structure of interest only. In grey the surface of the entire brain, in yellow the surface of the structure/region of interest and in purple all areas inside that structure/region that are significantly different between the genotypes at 0.1% FDR. On the right is a coronal slice through the average MR brain image. All colored regions indicate areas where the relative volume is significantly different between the 4 genotypes at 0.1% FDR. Cohort used included 8 *Del/+*, 10 wt, 11 *Del/Dup*, and 8 *Dup/+* animals.

In addition to neuropsychiatric features, over 50% of patients with 17q21.31 microdeletion also present with various brain structure changes[4, 5, 15]. Furthermore, 50% of patients with the 17q21.31 microdeletion present with microcephaly[8]. To identify potential morphological alterations of brain regions, we analyzed the brain structure of 8 *Del/+*, 10 wt, 11 *Del/Dup*, and 8 *Dup/+* mice using magnetic resonance imaging (MRI). Overall, we found significant differences in total brain volume between the genotypes (F_(3, 33)_ = 14.14, *p* < 0.001; *Del/+*: 458±23 mm^3^, wt: 448±10 mm^3^, *Del/Dup*: 446±12 mm^3^, *Dup/+*: 412±13 mm^3^, brain volumes given as mean±sd). *Dup/+* animals showed a globally reduced brain volume in comparison with that of the other genotypes. Using a segmented atlas that divides the brain into 159 separate brain regions[23–25], we examined the 83 structures of at least 1 mm^3^ in size. A reduction of the whole brain volume was noticed for *Dup/+* animals (Fig 3a). Brain structures significantly affected after a correction for multiple testing included the hippocampus, amygdala, nucleus accumbens, cingulate complex, entorhinal cortex, frontal region, and perirhinal cortex. Notably, for the majority of these structures, we observed opposite absolute volume changes in *Del/+* and *Dup/+* animals in comparison with values determined in wt littermates (Fig 3b). Relative volumes of the discussed regions are represented in the supplementary information (S3 Table).

### Effects of the *Arf2-Kansl1* dosage on synaptic transmission and synaptic plasticity in hippocampal slices

To explore if genes from the *Arf2-Kansl1* region regulate electrophysiological parameters in mouse neurons as suggested by changes in ChIP-seq profiles, we assessed basal synaptic transmission and synaptic plasticity by measuring field excitatory postsynaptic potentials (fEPSPs) in acute hippocampal slices from *Del/+* and *Dup/+* mice. In *Del/+* mutants, we observed decreased fEPSP slopes in mutant slices, especially in response to higher stimulus strengths (S6a Fig). Mean slopes of fEPSPs invoked by the maximum stimulus strength (4.2 V) were significantly smaller in slices from *Del/+* mice (1.46 ± 0.09 mV/ms) than wt littermates (1.87 ± 0.09 mV; F_(1,13.34)_ = 8.31; *P* = 0.025; two-way nested ANOVA, genotype effect). The mean paired-pulse ratio of slopes of fEPSPs evoked at a 50 ms interpulse interval was also significantly lower in mutant slices (S6c Fig; F_(1, 11.04)_ = 6.506; *P* = 0.027. No significant changes in LTP elicited by theta-burst stimulation were noted in slices from *Del/+* mice (S6 Fig).

Basal synaptic strength was slightly enhanced in slices from *Dup/+* mice, as fEPSPmax mean slope was nominally higher in slices from *Dup*/+ mice (2.12 ± 0.09 mV/ms) than in slices from wt littermates (1.89 ± 0.09 mV/ms; S6b Fig). However, the effect did not reach statistical significance (F_(1,8.67)_ = 3.09; *P* = 0.114; two-way nested ANOVA, genotype effect). Likewise, paired-pulse facilitation (S6d Fig) and LTP were not significantly different in slices from *Dupl*/+ and litter-matched wt mice (S6f Fig).

### Mice carrying a heterozygous deletion of *Kansl1* recapitulate the majority of phenotypes observed in animals carrying the *Arf2-Kansl1* deletion

**Fig 4:**
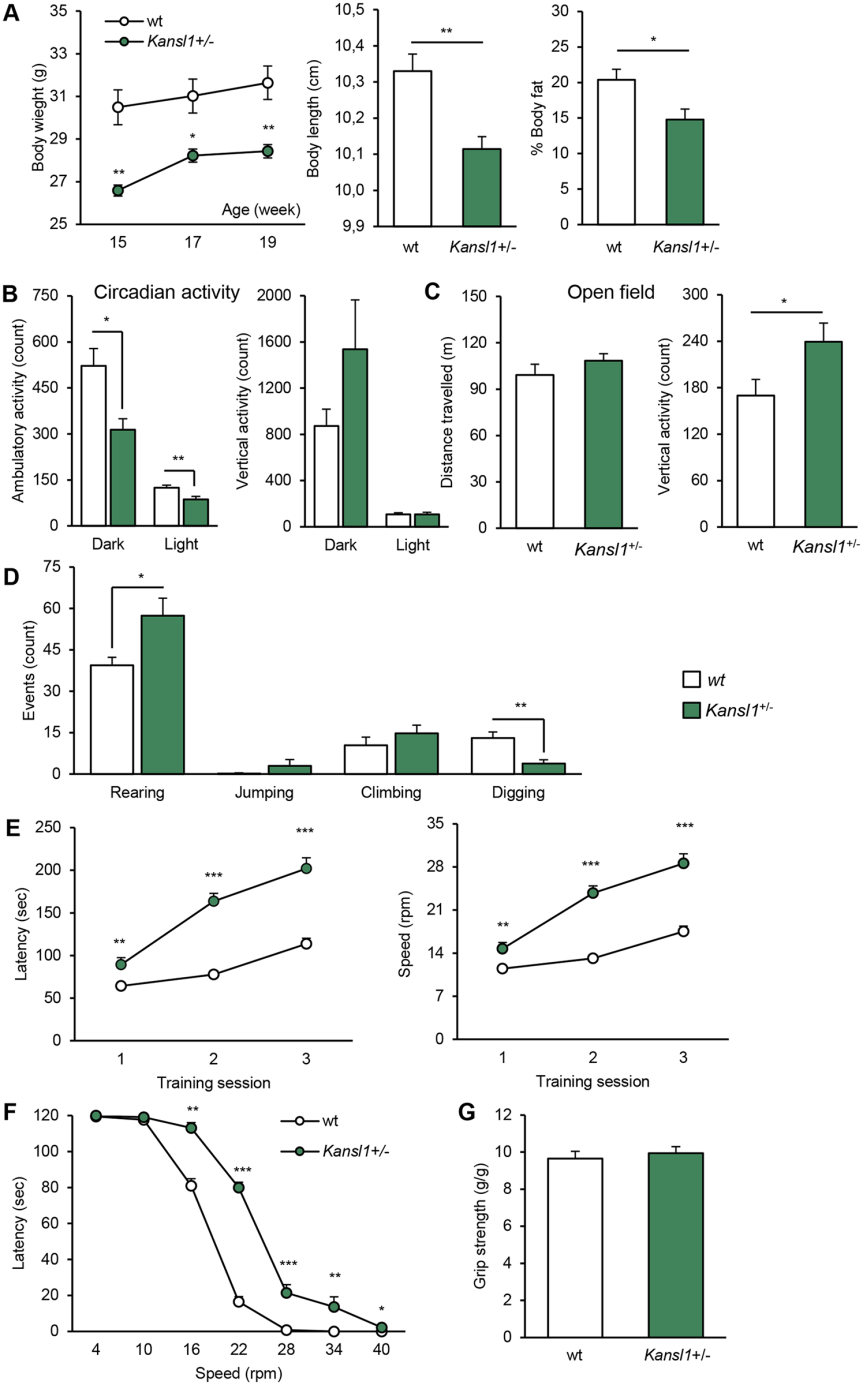
General behavioral characterization of *Kansl1*^*+/-*^ cohorts. **(A)** Body weight (g) of adult animals at 15, 17 and 19 weeks of age with Body length (distance from snout to tail basis) and Body fat percentage measured by qNMR of 20-week-old animals. Compared to wt littermates, *Kansl1*^+/-^ animals show body weight, size and adiposity deficits. **(B)** Circadian activity test. Graphs plot the spontaneous ambulatory activity (count) and the vertical activity/rears (count) during dark and light phases. **(C)** Open field test. Distance travelled (m), and vertical activity/rears (count) over 30 min of testing. **(D)** Repetitive behaviors. Graph plots occurences of rearing, jumping, climbing and digging behaviors (count) during 10 min of observation in a novel home cage. *Kansl1*^*+/-*^ animals show increase of rearing and decrease of digging levels reflecting an alteration of exploration activity. (**E**) Results are expressed as the time (s) that mice remained on an accelerating rod before falling during the training phase over 3 consecutive days of the rotarod test. (**F**) Corresponding rotational velocity (rpm) at the time of falling during the challenge phase of the rotarod test. The graph plots the time (s) that mice stayed on the rod when tested at constant speeds between 4 and 40 rpm. (**D**) Four-paw grip test. The conclusion of the rotarod and grip tests is that *Kansl1*^*+/-*^ animals show locomotor coordination improvements without alterations of muscular strenght. Graphs depict mean + s.e.m.. (**B-D**) Student’s t-test, *P < 0.05, **P < 0.01. (**A, E-G**) Repeated Measures ANOVA "genotype" analysis, Tukey's test, *P < 0.05, **P < 0.01, ***P < 0.001.

We performed similar examinations of mice with heterozygous ablation of *Kansl1* (*Kansl1*^+/-^ mice) in the same B6N genetic background and compared the outcome with the *Del/+* phenotypes.). In comparison with wt mice, *Kansl1*^+/-^ adult animals were underweight (two-way ANOVA genotype effect F_(1,30)_ = 11.729, *P* = 0.004; Fig 4a) *Kansl1*^+/-^ mice had a significantly smaller body size (F_(1,15)_ = 11.516, *P* = 0.004) and lower adiposity level (F_(1,15)_ = 6.813, *P* = 0.020; Fig 4a) than wt littermates in 20 week old animals. In aggregate, these data indicate many similarities in basic traits between the *Kansl1*^+/-^ and the *Del/+* carriers.

**Fig 5:**
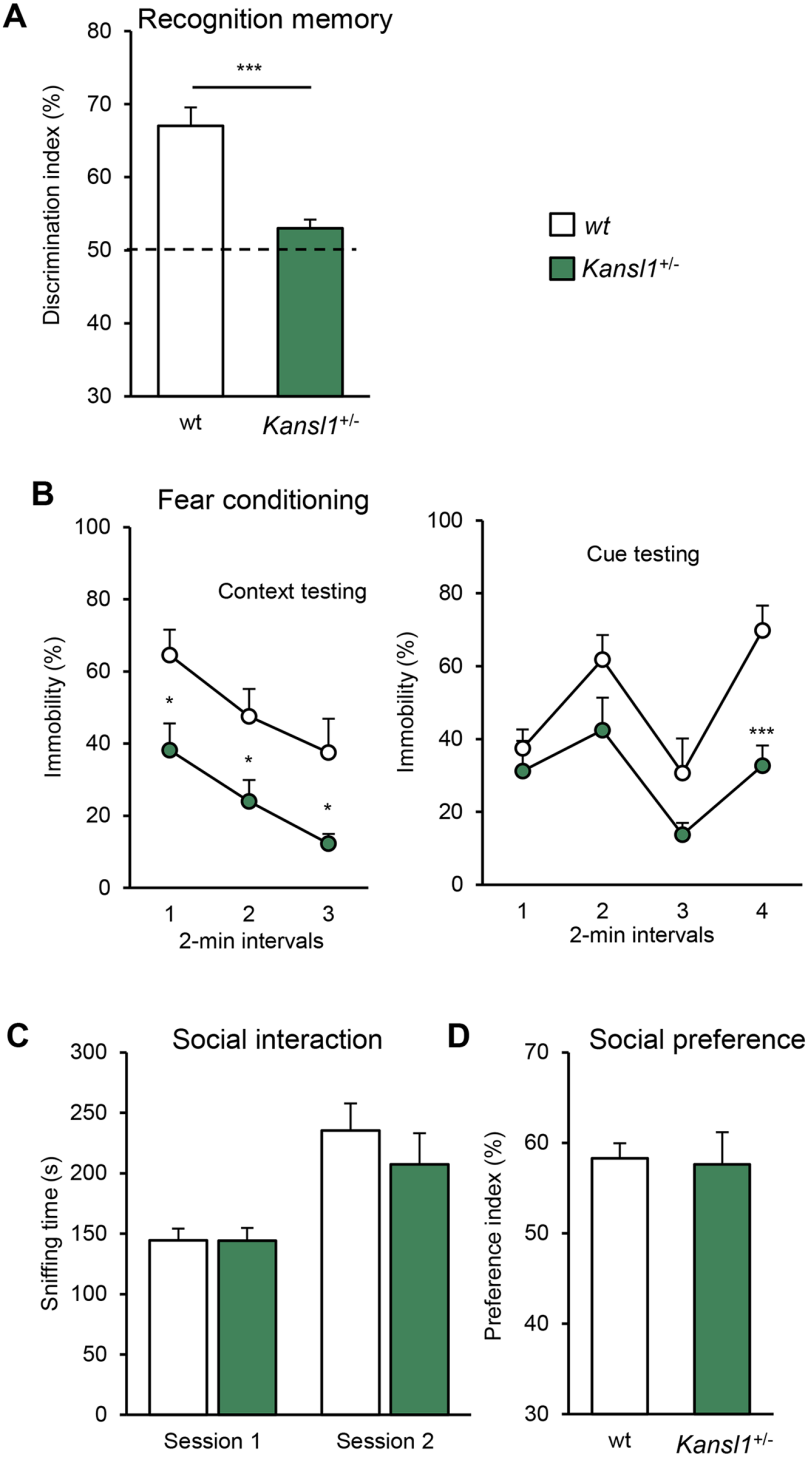
Learning and memory phenotypes in the *Kansl1*^*+/-*^. **(A)** Novel object recognition test. Discrimination index was calculated as the ratio of time spent exploring the novel object vs the familiar object in the choice trial after a 3 h retention delay. **(B)** Fear conditioning test. Plots represent the fraction of time spent freezing during test sessions. As above the 6-min context session was run 24 h after conditioning and the 8-min cue session was performed 5h after the context. A sequence of 2-min with no cue and 2 min with light/auditory conditioning stimulus was repeated two times. **(C-D)** Three-chamber sociability test. **(C)** Exploration time (s) of the first congener in the social interest session (session 1) and total exploration time of familiar and novel congeners in the social discrimination session (session 2). No delay was used between the two sessions. **(D)** Discrimination index was calculated as the ratio of time spent exploring the novel congener vs the familiar one. Data are represented as the mean ± s.e.m.. Cohort used included 8 *Kansl1*^*+/-*^ and 10 wt animals. Tukey's and Mann Whitney U tests following a significant one-way ANOVA and Kruskal Wallis analysis, respectively. *P < 0.05 vs wt, **P < 0.01 vs wt, ***P < 0.001 vs wt, ^#^ *P* < 0.05 vs all other groups.

Next, we examined the behavior of *Kansl1*^+/-^ mice. In a circadian activity test, *Kansl1*^*+/-*^ mice displayed normal patterns of activity (S7 Fig). However their baseline locomotor activity levels differed from those of wt littermates during the dark phase (F_(1,16)_ = 8.482, *P* = 0.010) and the light phase (F_(1,16)_ = 8.573, *P* = 0.010; Fig 4b). In the novel open field arena, *Kansl1*^*+/-*^ mice demonstrated an increased level of rearing behavior (F_(1,15)_ = 4.846, *P* = 0.044: Fig 4c). To investigate further this hyperactivity, we performed visual observations of animals in odorless home-cages (Fig 4d). Mutant mice displayed more intensive rearing behavior (F_(1,15)_ = 7.207, *p* = 0.017) and also showed a decreased level of digging behavior (F_(1,15)_ = 12.268, *p* = 0.003) in comparison with wt littermates. These results indicate a global alteration of *Kansl1*^*+/-*^ activity characterized, in particular, by locomotor hypoactivity and vertical hyperactivity. During the learning phase of the rotarod test, *Kansl1*^*+/-*^ mice displayed higher levels of motor coordination and learning than wt mice (two-way ANOVA genotype effect F_(1,30)_ = 115.867, *P* < 0.001; Fig 4e). In the test phase, *Kansl1*^*+/-*^ mice showed improvements for speed higher than 10rpm (Fig 4f), a phenotype not observed in the deletion due to lower power of the tests. Recognition memory was assessed in mice by using the novel object recognition task with a retention delay of 3 h. While no difference was observed in the acquisition session (S4 Table), *Kansl1*^*+/-*^ mice displayed a significant memory impairment compared to wt during the choice session (F_(1,15)_ = 22.566, *P* < 0.001; Fig 5a). Then, we evaluated associative memory with the fear conditioning test. No differences in the baseline and post-shock freezing levels were detected between *Kansl1*^*+/-*^ mice and wt littermates in the conditioning session (Fig 5b; S4 Table). In the context session, *Kansl1*^*+/-*^ mice displayed a lower incidence of freezing than wt littermates (H_(1,16)_ = 6.419, *P* = 0.011). In the cue session, a decreased freezing level was detected in *Kansl1*^*+/-*^ mice during the second 2-min cue period (F_(1,16)_ = 16.748, *P* < 0.001). Finally, we evaluated animal social behaviors with the three-chamber sociability test and the social interaction test (Fig 5c and 5d; S8 Fig). In both tests, no differences were observed between *Kansl1*^*+/-*^ mice and wt littermates.

### Epigenetic profiling in KdVS models identifies changes in regulation of neuronal processes and synaptic activity

**Fig 6:**
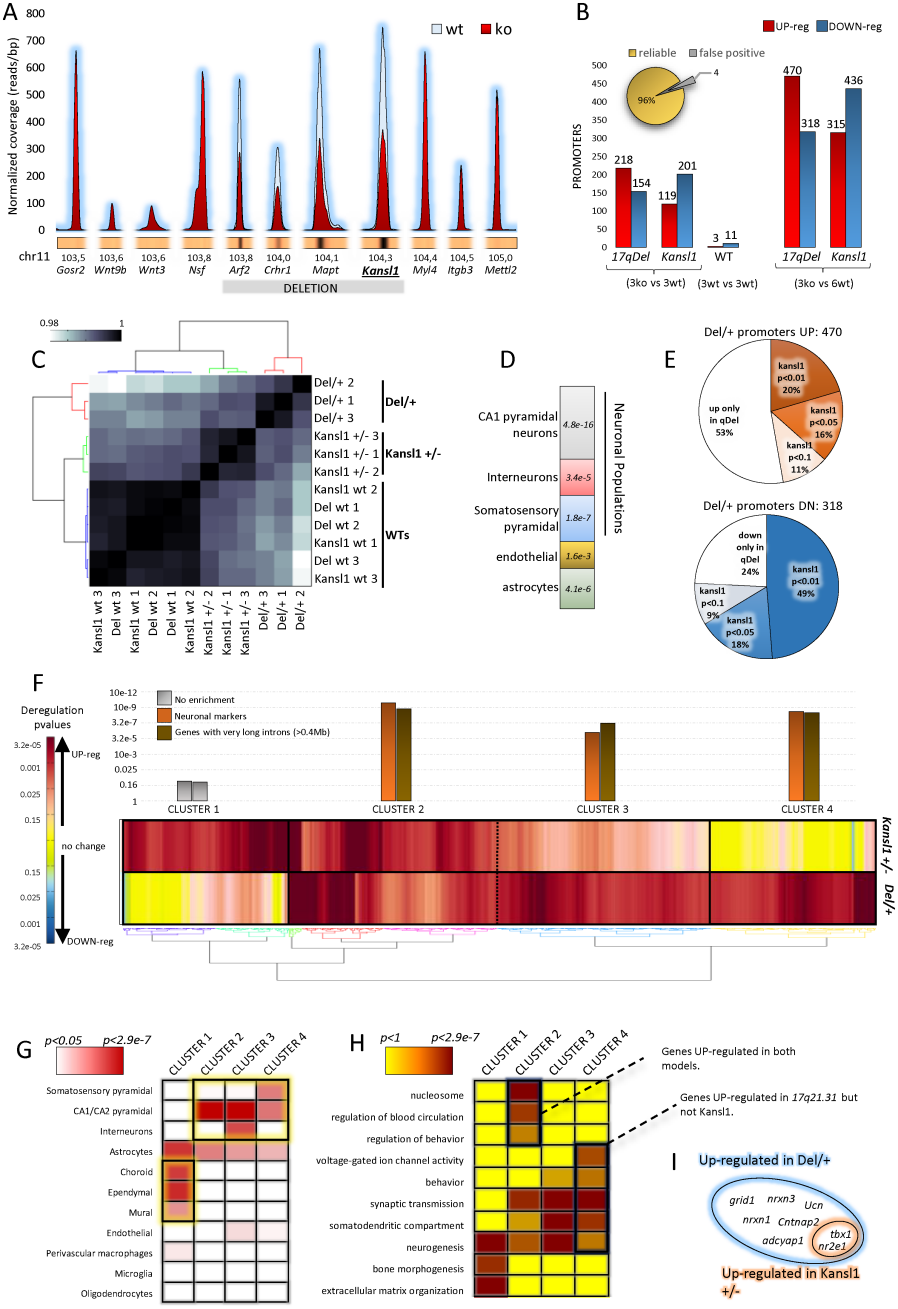
Epigenetic profiling in the hippocampus of KdV mouse models demonstrated non-neuronal and neuronal cell-specific alterations. (**A**) H3K4me3 pattern in the deleted region and its flanking sequences. Graph with a schematic representation of H3k4me3 peaks (averaged over three biological replicates) at the promoters of the genes in the 17q21.31 deleted region and flanking regions. Peaks within the deletion show half intensity as compared to wt, while peaks in the surrounding regions show the same intensity and patterning, thus confirming the absence of nonspecific-effects. (**B**) Overview of promoter deregulations. For each mouse model (*Kansl1*^*+/-*^ or *Del/+*) 3 wt biological replicas and 3 mutant biological replicas were generated for H3K4me3 ChIP-seq. DEseq2 algorithm was used to call the significant deregulations (*P* value<0.01) in the promoters. False positives, as detected by comparing the two groups of WT samples, can be estimated to only 4%. Given that no consistent changes were found among WT sets we opted to collapse all the 6 wts in one group and use it as the common control for both *Kansl1*^*+/-*^ and *Del/+* samples, thus increasing the sensitivity and detecting more dysregulation (right histograms) (**C**) Clustering of Pearson correlation calculated over the total dysregulated promoters clearly segregates the different conditions. (**D**) Cell types of *Del/+* up-regulated genes. The Stacked histogram represents the enrichment of cell-type specific markers in the *Del/+* UP-regulated genes. The strongest enrichment is for CA1 pyramidal neurons markers, followed by genes specific to somatosensory cortex pyramidal neurons, astrocytes and interneurons. A milder, but still significant, enrichment was found for endothelial cells. Together, these results suggest that *Del/+* deletion causes perturbations that are cell-type specific. (**E**) Overview of *Del/+* misregulation in the *Kansl1*^*+/-*^ model. Pie charts summarize the behavior of *Del/+* deregulated genes in the *Kansl1*^*+/-*^ model to evaluate the extent to which *Kansl1* alone is able to mimic the *Del/+* misregulations. To better describe the shades of gray always inherent to the *P* values, we classified the strength of *Kansl1*^*+/-*^ deregulation in four classes, ranging from no-change to the same intensity of *Del/+*. We could not detect any ‘reversal’ in the deregulations (i.e. genes up-regulated in one mutant being down-regulated in the other and vice versa) (**E**) Clustering of the *Del/*+ and *Kansl1*^*+/-*^ deregulated genes. The heat map includes all the genes that are significantly UP-regulated in either of the models. Enrichment of neuronal markers are based on published work. (**F**) Study of cell-type markers. The heat map represents the significance of the enrichments for specific markers in the 4 different clusters of section E. (**G**) Study of Cluster GO enrichments. The heat map represents the significance of the GO enrichments in the 4 clusters of section E. (**I**) overview of social behavior genes.

To identify potential gene expression differences in KdVS models, we carried out epigenetic profiling in the hippocampus, a brain region implicated in learning and memory processes, isolated from 3 *Del/+*, 3 *Kansl1*^*+/-*^ and 6 wt (3+3 matched littermates). We performed ChIP-Seq of H3K4me3 which is a histone mark located in actively expressed genes. As expected, H3K4me3 marks were lower in the *Arf2-Kansl1* region with half peak height values for *Arf2*, *Crhr1*, *Mapt* and *Kansl1* in the *Del/+* samples compared to those observed in wt samples and for the neighboring genes (Fig 6a). Analysis of H3K4me3 tracks (DESeq2, *p<0*.*01*) revealed 788 and 751 misregulated promoters in *Del/+* and *Kansl1*^*+/-*^, respectively (Fig 6b). Clustering of Pearson correlations, an unbiased method to measure the degree of similarity between large data sets, showed clear segregation between conditions and high concordance of biological replicates (Fig 6c; data are available in S5 to S10 Tables).

Cell-type marker analysis (Fig 6d; see Methods)[26] revealed that up-regulated promoters observed in *Del/+* mostly corresponded to genes expressed in neuronal populations (pyramidal neurons and interneurons). Furthermore, Gene Ontology (GO) analysis revealed that they present significant enrichments for the synapse (*p<7*.*9e-20*), and dendrite compartments (*p<3e-14*) as well as synaptic transmission (*p<9*.*1e-24*) or neurogenesis processes (*p<1*.*3e-22*; S12 and S20 Tables). In contrast, the term oxidoreductase and mitochondrion were found enriched respectively in *Del/+* and *Kansl1/+* down-regulated promoters using DAVID [27] but no other GO significant enrichments were found for *Del/+* (S11 and S19 Tables).

Of the 470 promoters up-regulated in *Del/+*, 36% (172) were also up-regulated in *Kansl1*^*+/-*^, whereas the two genotypes shared 67% (211) of genes that were down-regulated (Fig 6e). Among all the promoters up-regulated in either of the genetic conditions, we observed 4 distinct clusters of genes (Fig 6f). For cluster 1, 160 genes enriched in non-neuronal populations (astrocytes, ependymocytes, choroid plexus, mural cells, Fig 6g) are up-regulated in the hippocampus of *Kansl1* heterozygotes but not of *Del/+* mice. Cluster 2 encompassed 214 genes up-regulated in both genetic conditions and enriched in markers of CA1/CA2 pyramidal neurons and astrocytes, while Cluster 3 contained 212 genes enriched in neuronal markers and whose expression was up-regulated to a greater extent in *Del/+*. Finally, we noted that cluster 4 comprised 162 genes upregulated specifically in *Del/+* and expressed in CA1/CA2 pyramidal neurons and astrocytes. Each cluster showed a specific GO enrichment profile (Fig 6h). Several neuronal processes, including synaptic transmission and neurogenesis were overrepresented GO terms in genes from clusters 2, 3 and 4. Cluster 2 genes (up in both models) were also enriched in DNA-packaging and nucleosomes (Fig 6h). In the *Del/+* hippocampi, 8 genes involved in social behavior were up-regulated and only 2 of these, *Tbx1* and *Nr2e1*, were also dysregulated in *Kansl1*^*+/-*^ mice (Fig 6i). These results suggest a dominance of *Del/*+ with respect to *Kansl1* for determining social behavior.

### Effects of the *Arf2-Kansl1* dosage on synaptic transmission and synaptic plasticity in hippocampal slices

To explore if genes from the *Arf2-Kansl1* region regulate electrophysiological parameters in mouse neurons as suggested by changes in ChIP-seq profiles, we assessed basal synaptic transmission and synaptic plasticity by measuring field excitatory postsynaptic potentials (fEPSPs) in acute hippocampal slices from *Del/+* and *Dup/+* mice. In *Del/+* mutants, we observed decreased fEPSP slopes in mutant slices, especially in response to higher stimulus strengths (S6 Fig). Mean slopes of fEPSPs invoked by the maximum stimulus strength (4.2 V) were significantly smaller in slices from *Del/+* mice (1.46 ± 0.09 mV/ms) than wt littermates (1.87 ± 0.09 mV; F_(1,13.34)_ = 8.31; *P* = 0.025; two-way nested ANOVA, genotype effect). The mean paired-pulse ratio of slopes of fEPSPs evoked at a 50 ms interpulse interval was also significantly lower in mutant slices (S6 Fig; F_(1, 11.04)_ = 6.506; *P* = 0.027. No significant changes in LTP elicited by theta-burst stimulation were noted in slices from *Del/+* mice (S6c–S6e Fig).

Basal synaptic strength was slightly enhanced in slices from *Dup/+* mice, as fEPSPmax mean slope was nominally higher in slices from *Dup*/+ mice (2.12 ± 0.09 mV/ms) than in slices from wt littermates (1.89 ± 0.09 mV/ms). However, the effect did not reach statistical significance (F_(1,8.67)_ = 3.09; *P* = 0.114; two-way nested ANOVA, genotype effect). Likewise, paired-pulse facilitation (S6 Fig) and LTP were not significantly different in slices from *Dupl*/+ and litter-matched wt mice (S6 Fig).

## Discussion

In this study, we described the first mouse models of Koolen-de Vries syndrome (KdVS) and 17q21.31 microduplication syndrome. *Del/+* mice showed similar phenotypes observed in KdVS patients: higher level of social interaction, lower level of recognition memory, associative learning and memory and brain malformations [3, 5, 15, 28]. We found a single phenotypic similarity between patients carrying the 17q21.31 microduplication and *Dup/+* mice, which is microcephaly that has been reported in 50% of the human individuals with this microduplication [8]. Several SNPs associated with risk for Alzheimer’s disease (AD) were identified near *MAPT* and *KANSL1* in humans and they appeared to be correlated with an overexpression of both genes in different brain regions [29]. This observation was further supported by the description of a familial form of late onset AD due to the microduplication of the 17q21.31 region [30]. Thus it would be important now to follow cognition in ageing cohorts carrying the Dup/+ allele generated here as young individuals analysed in the present study do not shown any cognitive impairment, but rather more some improvement in associative memory.

Overall, the phenotypic comparison observed in the *Del/+* and *Kansl1*^*+/-*^ models confirms the critical importance of *KANSL1* in KdVS[3, 15]. Nevertheless, increased social interaction was not found to be affected in *Kansl1* haploinsufficient mice whereas it is predominant in humans with *KANSL1* mutations [3, 15]. This discrepancy could reflect either different dosage threshold levels in the mouse and human that governs proper social interaction, with the mice needing more change to induce such friendly phenotype. Indeed we have found more genes associated with social behavior misregulated in the *Del/+* brain compared to the *Kansl1* heterozygotes. An educated guess would be that the haploinsufficiency of another gene(s) from the *Arf2-Kansl1* region would contribute to this phenotype in mouse.

The hippocampal epigenetic analysis of 17q21.31 models unraveled several features. Only the mitochondrion term was enriched in the *Kansl1/+* down-regulated genes, a situation partially similar to a recent study where KANSL1 and its partner MOF were observed in mitochondria regulating expression of genes involved in oxidative phosphorylation [31]. Interestingly the oxidoreductase term was enriched in *Del/+* down-regulated genes. Thus it would be interested to follow mitochondrial activity in the mouse models. Another common set of dysregulated promoters, were largely affecting CA1 neuronal populations and neuronal functioning and includes many genes with long introns. Common up-regulated genes also appear to be implicated in DNA-packaging and nucleosomes, possibly reflecting the outcome of the misregulated KANSL1 activity. Interestingly, several genes (*Adcyap1*, *Cntnap2*, *Grid1*, *Nrxn1*, *Nrxn3*, *Ucn*, *Tbx1*, *and Nr2e1*), that are associated with disorders [32, 33], stress response [34], social behaviors [35–37] or autism spectrum disorders [38–42], are up-regulated specifically in the hippocampus of *Del/+* mice. Deregulation of these genes may be a molecular underpinning of the friendly/amiable affect of 17q21.31 deletion patients. Expression of the majority of those genes (except for *Txb1* and *Nr2e1*) was not altered in *Kansl1*^*+/-*^ mice. We also emphasize that two overexpressed genes, *Ucn* and *Adcyap1*, and one underexpressed gene, *Chd1l*, found deregulated in *Del/+* mice are linked to corticotropin release and are associated with stress response. Those genes may be relevant for the overly friendly social phenotypes observed in 17q21.31 deletion carriers.

Electrophysiological experiments confirmed that dosage of one or several genes within the 17q21.31 syntenic region affects basal synaptic transmission and short-term plasticity of excitatory synapses in the hippocampus. Noted disturbances in the expression level of several genes could contribute to this impairment. For example, dysregulation of *Cntnap2* could affect migration of interneurons [37] and inhibitory synaptic function [43], which could, in turn, alter excitatory synaptic responses. Other gene affected by 17q21.31 mutations is Nrxn1 that shapes the balance between excitatory and inhibitory synaptic activity [44, 45]. Such a defect at the expression level may account for the change in synaptic strength and impaired learning and memory.

In conclusion, this study confirms a previously hypothesized role of *KANSL1* in the manifestation of KdVS phenotypes and extends substantially our knowledge about biological processes affected by these mutations. With these new genetic tools, we can explore the function of these genes and dissect further the pathophysiological mechanisms to eventually inform potential therapeutic avenues.

## Methods

### Ethical statement and mouse lines

The 17p21.31 mutant mice carrying the deletion of the *Arf2–Kansl1* region (noted *Del/+*), or the reciproqual duplication (noted *Dup/+*), were generated on the C57BL/6N genetic background (see Supplementary information). The *Kansl1*^*+/-*^ mutant mice were derived in a C57BL/6N genetic background from the unique IKMP ES cell clone HEPD0766_8_G02. *Kansl1*^*tm1b(EUCOMM)Hmgu*^[46] animals were obtained by breeding *Kansl1*^*tm1a(EUCOMM)Hmgu/+*^ mice with animals expressing the Cre recombinase[47] to generate the *Kansl1*^*tm1b(EUCOMM)Hmgu/+*^ (noted here *Kansl1*^*+/-*^). The local ethics committee, Com’Eth (n°17), approved all mouse experimental procedures, under the accreditation number 2012–069.

### Behavioral analysis

Behavioral studies were conducted in 12-20-week old animals. All assessments were scored blind to the genotype as recommended by the ARRIVE guidelines[48, 49]. All the experimental procedures for behavioral assessments have been described[50, 51] and are detailed in the supplementary information.

### Craniofacial and image acquisition

Craniofacial phenotyping is described in the supplementary data. Magnetic resonance imaging (MRI) was used to identify alterations of brain regions in 17q21.31-homologous CNV mice (8 *Del/+*, 10 wt, 11 *Del/Dup*, and 8 *Dup/+* mice). MRI scans were acquired from 41 male mice at 43 weeks of age with specimens prepared as described[52] and detailed with the image processing in the supplementary information.

### Hippocampal slice electrophysiology

Acute hippocampal slices were used to record field excitatory post synaptic potentials (fEPSPs), by using an electrophysiological suite of 8 MEA60 set-ups consisting of a MEA1060-BC pre-amplifier and a filter amplifier (gain 550×) (Multi Channel Systems, Reutlingen, Germany) as described[50, 53]. All experiments were performed using two-pathway stimulation of the Schaffer collateral/commissural fibers in the CA1 area of 350-μm thick hippocampal slices (see supplementary information).

### ChIP-seq

Adult hippocampi from *Del/+*, *Kansl1*^+/-^ and wt mice were dissected and snap-frozen in liquid nitrogen. Tissue samples were ground in a liquid-nitrogen chilled mortar and the resulting powder was used for ChIP. Chipping for H3K4me3 (Diagenode A5051-001P) was performed as in (www.blueprint-epigenome.eu/UserFiles/file/Protocols/Histone_ChIP_July2014.pdf). Libraries were synthetized with KAPA Hyper prep kit (KK8504) following the manufacturer’s instructions. The libraries were pooled (4/lane) and sequenced on the illumina HiSeq. Libraries were mapped with BWA (0.6.2). Peaks were called with a custom C++ script and DEseq2 (R+) was used to perform statistical comparisons. Data are deposited in GEO under accession GSE80311. All enrichment analyses are made from standard hypergeometric tests with Benjamini or Bonferroni correction. GO annotations are updated to 25/6/2015.

### Cell-types enrichments

ChIP-seq data and ChIP-seq supplementary tables were deposited in GEO and available at the link https://www.ncbi.nlm.nih.gov/geo/query/acc.cgi?token=cnavacaedzgztgz&acc=GSE80311. Cell-types enrichments are based on the single-cell RNAseq data from Amit Zeisel et al., 2015, “cell types in the mouse cortex and hippocampus revealed by single-cell rna-seq” [26]. In this work, single-cell RNAseq was used to measure trascriptomes of >3000 single cells, allowing to define the markers of 11 different cell types in adult (P21-P30) hippocampus and somatosensory cortex. Given that also our ChIP-seq is done on adult (P30) hippocampus, single-cell RNAseq data from Zeisel et al. becomes a highly valuable resource to gain insight at the cell type level. Here we performed standard hypergeometric tests with bonferroni correction against the cell-type markers derived from data of Zeisel et al. in order to evaluate the abundance of specific markers in deregulated gene sets. A significant enrichment (p<0.01) means that a high amount of markers of a specific cell type is found in *Kansl1*^*+/-*^ or *Del/+* deregulated genes, suggesting that the latter cell type should be particularly affected. The complete statistical data and the lists of markers found in de-regulated genes are fully available in S5 to S20 Tables.

### Statistical analyses for behavior and electrophysiology

All acquired behavioral data were analyzed using a one-way ANOVA analysis with a *post-hoc* Tukey’s test, when applicable, non-parametric Kruskal-Wallis test or Mann-Whitney *U* test using the Sigma Plot software (Ritme, France). The Pearson’s chi-squared test was used for mutant allele transmission. Data are represented as the mean ± s.e.m. and differences were considered to be significant if *P* < 0.05. When comparing freezing levels between wt, *Del/+*, and *Dup/+* animals over different time points during extinction we used the two-Way ANOVA Repeated Measures statistical test followed by Holm-Sidak post-hoc tests to evaluate for interactions between the groups. Otherwise, when comparing wt data with those obtained from respective *Del/+* or *Dup/+* animals for a single time point, we used Student’s t-test. When data did not follow a normal distribution, we used the Mann-Whitney rank-based statistical test.

In electrophysiological experiments input-output relationships were compared initially by a mixed model repeated-measures ANOVA and Bonferroni *post hoc* test implemented in Prism 5 (GraphPad Software, San Diego, USA) using individual slice data as independent observations. Since several slices were routinely recorded from every mouse, fEPSPmax, PPF and LTP values between wt and mutant mice were compared using two-way nested ANOVA design with genotype (group) and mice (sub-group) as fixed and random factors respectively (STATISTICA v.10, StatSoft, USA). DF error was computed using the Satterthwaite’s method and main genotype effect was considered significant if *P* < 0.05. Graph plots and normalization were performed using OriginPro 8.5 (OriginLab, Northampton, USA). Electrophysiological data are presented as the mean ± s.e.m. with *n* and *N* indicating number of slices and mice respectively.

## Supporting information

S1 Appendix Detailed materials and methods with additional references for supporting figures. (DOCX)

S1 Fig Spontaneous locomotor activity and feeding behavior of the *Del-Dup* cohort during the circadian activity test. Patterns of locomotor activity (**A**) and vertical activity (**B**) during the 32-hours of test. (**C-D**) Feeding behaviors. Water (**C**) and pellet (**D**) consumption during the 32-hours of testing. (**E**) Pellets lost by animals which passed through the holed ground. *Del/+*, *Dup/+*, and *Del/Dup* animals showed normal pattern of activity and consumption during the test. Data are represented as the mean + s.e.m..
(TIF)

S2 Fig Locomotor coordination and muscular capacities of the *Del-Dup* cohort. (**A-B**) Training phase of the rotarod test. (**A**) Results are expressed as the time (s) that mice remained on an accelerating rod (4–40 rpm over 5 min) before falling. (**B**) Corresponding rotational velocity (rpm) at the time of falling. (**C**) Challenge phase of the rotarod test. The graph plots the time (s) that mice stayed on the rod when tested at constant speeds between 4 and 40 rpm. (**D**) Four-paw grip test. We did not detect locomotor coordination or muscular defects in the mutant mice. Graphs depict mean + s.e.m..
(TIF)

S3 Fig Spatial learning and memory performances of the *Del-Dup* cohort in the Morris water maze (MWM) test. (**A-B**) Acquisition phase. (**A**) Distance travelled (m) to find the platform along acquisition (A1 –A6) and reversal (Cued1) sessions. (**B**) Corresponding latency (s) to find the platform. (**C-D**) Removal phase. Mice were scored for the percentage of time spent in the different quadrant of the MWM (**C**) and the annulus crossing counts (**D**) during one single trial where the platform was removed. *Del/+*, *Dup/+*, and *Del/Dup* animals do not show any learning and memory defects in the MWM test. All graphs depict mean + s.e.m..
(TIF)

S4 Fig Social behaviors of the *Del-Dup* cohort. (A-D) Three-chamber test. (**A, C**) Social interest session. (**B, D**) Social discrimination session. (**A-B**) Time (s) in chambers during the social interest session (**A**) in which restrictive area of chamber 1 contains an unfamiliar mouse and during the social discrimination session (**B**) where chamber 1 and 2 restrictive areas contain respectively familiar and unfamiliar mice. (**C-D**) Corresponding time (s) spent to explore restrictive areas. Compared to wt littermates, *Del/+* animals spent more time to interact with the stranger mouse during the social interest session. All genotypes show similar social discrimination capacities. (**E-G**) Social interaction test. Latency to first contact (**E**), sniffing time (**F**) and following time (**G**) (s) of 2 animals of similar genotype from different house cages putting together in an open-field during 10 min. All graphs depict mean + s.e.m.. Tukey's test following a significant one-way ANOVA. *P < 0.05 vs wt.
(TIF)

S5 Fig Craniofacial analysis of the *Del-Dup* cohort. **(A)** Representative reconstructed 3D skull images with localisation of the 39 landmarks used in the study. Euclidian distances between the different landmarks allowed calculation of both the form (or size) difference (FD) and the shape difference (SD). Analysis revealed no defect of skull size in *Del/+* **(B)** and *Dup/+* **(D)** animals. Trends for skull shape alteration of *Del/+* **(C)** and *Dup/+* **(E)** mice were noticed. **(F)** Principal component analysis based on the 39 landmarks and symmetric component captured in the study using the MorphoJ software. The analysis allowed to segregate the 3 genotypes based on 3 main component PC1, PC2 and PC3 that affect the skull shape as showed by wireframe graphs with the shape of wt control in light blue and the shape associated with the component in dark blue. Landmarks are showed in red.
(TIF)

S6 Fig Effect of the *Arf2-Kansl1* dosage on electrophysiological parameters measured in the CA1 area of hippocampal slices. **(A–B)** Basal synaptic transmission. **(A)** Input-output relationships illustrate averaged fEPSP slopes in slices from *Del/+* (*n* = 28; *N* = 9) and wt mice (*n* = 33; *N* = 8) in response to stimulation of Schäffer collaterals by biphasic voltage pulses of 0.1–4.2 V. Representative families of fEPSP traces are given on the right side. Synaptic responses were significantly lower in *Del/+* mice (F_(9, 531)_ = 7.59; *P* < 0.0001). Mean slopes of fEPSPs invoked by the maximum stimulus strength (4.2 V) were significantly smaller in slices from *Del/+* mice (1.46 ± 0.09 mV/ms) than wt littermates (1.87 ± 0.09 mV; F_(1,13.34)_ = 8.31; *P* = 0.025; two-way nested ANOVA, genotype effect). **(B)** Input-output relationships illustrate averaged fEPSP slopes in slices from *Dup/+* (*n* = 27; *N* = 9) and wt mice (*n* = 27; *N* = 10). Basal synaptic transmission was nominally enhanced in *Dup/+* mice: fEPSPmax mean slope was nominally higher in slices from *Dup*/+ mice (2.12 ± 0.09 mV/ms) than in slices from wt littermates (1.89 ± 0.09 mV/ms) but, the effect was not statistically significant (F_(1,8.67)_ = 3.09; *P* = 0.114; two-way nested ANOVA, genotype effect). **(C–D)** Paired-pulse facilitation. **(C)** Paired-pulse facilitation was slightly but significantly lower in *Del/+* animals (*n* = 28; *N* = 9) than in wt littermates (*n* = 33; *N* = 8; F_(1, 11.04)_ = 6.506; *P* = 0.027). Representative fEPSP sweeps are presented on the right side. **(D)** Paired-pulse facilitation was nominally higher in *Dup/+* animals (*n* = 27; *N* = 9) than wt littermates (*n* = 27; *N* = 10), but the effect did not reach statistical significance (F_(1, 4.92)_ = 5.402; *P* = 0.069). **(E–F)** Theta-burst stimulation elicited pathway-specific long-term potentiation (LTP) of synaptic transmission in hippocampal CA1 area. **(E)** Normalized magnitude of LTP 60–65 min after LTP induction was similar in *Del/+* mice (163 ± 4%; *n* = 27; *N* = 9; *P* = 0.88) and in their wt counterparts (165 ± 8%; *n* = 33; *N* = 8). Examples of test pathway fEPSP traces immediately before and 1 h after theta-burst stimulation are given on the right side. **(F)** Normalized magnitude of LTP 60–65 min after LTP induction did not differ in *Dup/+* mice (166 ± 7%; *n* = 27; *N* = 9; *P* = 0.29) relatively to their wt counterparts (154 ± 6%; *n* = 26; *N* = 10). Data are expressed as the mean ± s.e.m.
(TIF)

S7 Fig Spontaneous locomotor activity and feeding behavior of the *Kansl1*^*+/-*^ cohort during the circadian activity test. Patterns of locomotor activity (**A**) and vertical activity (**B**) during the 32-hours of test. (**C-D**) Feeding behaviors. Water (**C**) and pellet (**D**) consumption during the 32-hours of testing. (**E**) Pellets lost by animals which passed through the holed ground. Data are represented as the mean + s.e.m..
(TIF)

S8 Fig Social behaviors of the *Kansl1*^*+/-*^ cohort. (**A-D**) Three-chamber test. (**A, C**) Social interest session. (**B, D**) Social discrimination session. (**A-B**) Time (s) in chambers during the social interest session (**A**) in which restrictive area of chamber 1 contains an unfamiliar mouse and during the social discrimination session (**B**) where chamber 1 and 2 restrictive areas contain respectively familiar and unfamiliar mice. (**C-D**) Corresponding time (s) spent to explore restrictive areas. Compared to wt littermates, *Del/+* animals spent more time to interact with the stranger mouse during the social interest session. All genotypes show similar social discrimination capacities. (**E-G**) Social interaction test. Latency to first contact (**E**), sniffing time (**F**) and following time (**G**) (s) of 2 animals of similar genotype from different house cages putting together in an open-field during 10 min. The conclusion of the social behavior tests is that *Kansl1*^*+/-*^ animals do not show social alterations. All graphs depict mean + s.e.m..
(TIF)

S1 Table Activity and anxiety characterization of the *Del-Dup* cohort. In the circadian activity test, both Del/+ and Dup/+ animals showed global vertical hypoactivity. *Del/+* mice showed locomotor hypoactivity during dark and light phases and rearing hypoactivity during the light phase. *Dup/+* mice showed rearing hypoactivity during habituation phase. No alteration of feeding behavior was noticed during the test. During open field sessions, *Del/+* and *Dup/+* mice showed respectively trends rearing hyperactivity and hypoactivity. No phenotype was observed in the elevated plus maze test. No difference in activity and anxiety was observed between *Del/Dup* mice and wt littermates. Data are mean ± SEM.
(DOCX)

S2 Table Behavioral characterization of the *Del-Dup* cohort. No phenotype was observed in the Y maze test. Object recognition memory of mice was assessed with a retention delay of 3 h. In the first session (S1) of test, no difference in object exploration was noticed. In the retention session (S2), *Del/+* mice showed recognition memory deficits. In the Morris water maze, no difference of spatial learning and memory was observed between the different genotypes. Mutant mice and wild-type littermates travelled the same distance and needed the same time to find the platform from first day to sixth day (D6) of acquisition. In the probe test (PT) on day 7, when the platform is removed, all mice spent a similar percentage of time in the target quadrant. In the fear conditioning test, all genotypes displayed similar level of activity in the conditioning session before footshock. Post-choc freezing was decreased in *Del/+* and *Del/Dup* mice without significance. In the 6-min contextual session, *Dup/+* animals showed a global higher freezing without significance. In cue sessions, *Del/+* and *Dup/+* mice displayed respectively lower and higher level of freezing in comparison with wt littermates. In the three-chamber sociability test, *Del/+* mice showed an increased level of social interaction with the first stranger in first session (S1). *Del/+* mice also presented trends for higher level of social interaction with the first stranger and the second stranger in the second session (S2). No alteration of social preference was found between genotypes. In the social interaction test, *Del/+* animals displayed trends for shorter first contact latency and higher level of social interaction whereas *Dup/+* mice displayed trends for longer first contact latency and lower level of social interaction in comparison with wt littermates. No phenotype was observed in rotarod and grip tests. *Del/Dup* mice showed similar performances than wt littermates. Data are mean ± SEM.
(DOCX)

S3 Table Comparison of the absolute volumes of 83 brain structures observed in mutant *Del/+* and *Dup/+* animals with wt littermates by magnetic resonance imaging. (DOCX)

S4 Table Behavioral characterization of *Kansl1*^+/-^ cohorts. In the circadian activity test, *Kansl1*^*+/-*^ mice showed locomotion hypoactivity in dark and light phases. No alteration of feeding behavior was noticed during the test. During open field sessions, *Kansl1*^*+/-*^ animals showed rearing hyperactivity. Observation of repetitive behaviors in odorless home-cages revealed decreased level of digging and increased level of rearing in *Kansl1*^*+/-*^ mice in comparison with wt littermates. Object recognition memory of mice was assessed with a retention delay of 3 h. In the first session (S1) of test, no difference in object exploration was noticed. In the retention session (S2), *Kansl1*^*+/-*^ mice showed potent recognition memory deficits. In the fear conditioning test, no difference of activity in the conditioning session before footshock was observed between *Kansl1*^*+/-*^ mice and wt littermates. In the 6-min contextual session, *Kansl1*^*+/-*^ mice showed a reduced level of freezing. In cue sessions, *Kansl1*^*+/-*^ mice also showed reduced level of freezing with significant statistical difference during the second cue. During the rotarod test, *Kansl1*^*+/-*^ mice displayed an important improvement of motor coordination from the first day (D1) to the third day (D3) of test. No phenotype was observed in the grip test. Data are mean ± SEM.
(DOCX)

S5 Table Cluster1 GO enrichments. (TXT)

S6 Table Cluster2 GO enrichments. (TXT)

S7 Table Cluster3 GO enrichments. (TXT)

S8 Table Cluster4 GO enrichments. (TXT)

S9 Table *Kansl1*^*+/-*^ DOWN-reg GO enrichments. (TXT)

S10 Table *Kansl1*^*+/-*^ UP-reg GO enrichments. (TXT)

S11 Table *Del/+* DOWN-reg GO enrichments. (TXT)

S12 Table *Del/+* UP-reg GO enrichments. (TXT)

S13 Table Cluster1 other enrichments (cell-types, chromosomes, gene features,…). (XLSX)

S14 Table Cluster2 other enrichments (cell-types, chromosomes, gene features,…). (XLSX)

S15 Table Cluster3 other enrichments (cell-types, chromosomes, gene features,…). (XLSX)

S16 Table Cluster4 other enrichments (cell-types, chromosomes, gene features,…). (XLSX)

S17 Table *Kansl1*^*+/—*^DOWN-reg other enrichments (cell-types, chromosomes, gene features, …). (XLSX)

S18 Table *Kansl1*^*+/-*^ UP-reg other enrichments (cell-types, chromosomes, gene features, …). (XLSX)

S19 Table *Del/+* DOWN-reg other enrichments (cell-types, chromosomes, gene features, …). (XLSX)

S20 Table *Del/+* UP-reg other enrichments (cell-types, chromosomes, gene features, …). (XLSX)
