# Supplementary material for: Mouse models of 17q21.31 microdeletion and microduplication syndromes highlight the importance of Kansl1 for cognition
Source: PLoS Genet. 2017 Jul 13;13(7):e1006886. doi: 10.1371/journal.pgen.1006886 (PMC5531616; doi:10.1371/journal.pgen.1006886)
Supplement: S1 Appendix — (DOCX) [file pgen.1006886.s001.docx]

## S1 Appendix . Detailed Materials and Methods

## Ethical statement and generation of mice carrying rearrangements of the *Arf2-Kansl1* genetic interval

All experiments were performed in accordance with the Directive of the European Parliament: 2010/63/EU, revising/replacing Directive 86/609/EEC and with French Law (Decret n° 2013-118 01 and its supporting annexes entered into legislation 01February 2013) relative to the protection of animals used in scientific experimentation. YH was the principal investigator of this study (accreditation 67-369) in our animal facility (Agreement C67-218-40). The mouse lines are available through the INFRAFRONTIER/European Mouse Mutant Archive (EM:06133 and EM:06134) or the International Mouse Phenotyping Consortium ([www.mousephenotype.org](http://www.mousephenotype.org)) for *Kansl1*.

To generate mouse models of the 17q21.31 rearrangements, we introduced LoxP sites flanking the syntenic region located on chromosome 11E1 **(**Fig. 1a). Mutant mice were obtained by using an *in vivo* TAMERE strategy [1, 2]. LoxP sites were first introduced by homologous recombination in C57BL/6N (B6N) embryonic stem cells at *Sult1a1* and *Spn* loci in the same orientation and the corresponding mouse line were generated. Selection cassettes were excised and mice were crossed with *Hprt<tm1(cre)Mnn>* mice [3], expressing the Cre recombinase under the control of the X-linked hypoxanthine guanine phosphoribosyl transferase gene promoter active in oocytes. Females born from this mating and bearing both the *Hprt<tm1(cre)Mnn>* transgene and loxP sites in a trans configuration were mated with wt B6N males (Supp Fig. 1B). We recovered mice carrying the deletion (*Del/+*) and the duplication (*Dup/+*) for the *Arf2-Kansl1* region with a recombination frequency of respectively 11 and 7 animals out of 140 newborns. Deletion of the *Arf2–Kansl1* region was identified by PCR using primers Fwd1 (5’-TCCTAACCCACGGGTCAGCCTA-3’) and Rev2 (5’-GCCCATACGGATGTTTCCTTCCAA-3’). Duplication of the same region was identified using primers Fwd2 (5’- CACCACGGGAGCAAGCAACTGAG-3’) and Rev1 (5’-CCAGCCCACTCAGACTTTCCAGAAT-3’). The wt allele was identified using Fwd1 and Rev1 primers. PCR reactions amplified specific products from deletion, duplications and wt alleles of 448 bp, 653 bp and 341 bp, respectively (Fig. 1C). All mice were genotyped by PCR using the following program: 95 °C /5 min; 35× (95 °C/30 s, 65 °C/30 s, 70 °C/1 min), 70 °C/5 min.

Deletion of the targeted exon in *Kansl1^tm1a(EUCOMM)Hmgu/+^* was identified using primers FwdRi1F (5’-GCACATGGCTGAATATCGACGGT-3’) and RwdLxr (5’-ACTGATGGCGAGCTCAGACCATAAC-3’) to generate the *Kansl1^tm1b(EUCOMM)Hmgu/+^*. PCR reactions produced a 471 bp amplicon using the following program: 95 °C /4 min; 35x (94 °C/30 s, 62 °C/30 s, 72 °C/1 min), 72 °C/7 min.

## Behavioral analysis

To characterize the mice carrying both the deletion and the duplication, we crossed *Del/+* with *Dup/+* animals. We used the *Del/Dup* as pseudo-disomic mice to investigate effects that are not dependent on the copy number of the region but could be generated by the impact of the rearrangement on the transcription of neighbouring genes. We generated experimental animal cohorts by selecting mice from litters containing a minimum of two male pups.

To study behaviour of animals, we crossed *Del/+* with *Dup/+* animals and generated two *Del-Dup* cohorts of males with in total 18 *Del/+*, 24 wt, 11 *Del/Dup*, and 11 *Dup/+* animals. After weaning, animals were sorted by litters into 39 x 20 x 16 cm cages (Green Line, Techniplast, Italy) where they had free access to water and food (D04 chow diet, Safe, Augy, France). The temperature was maintained at 23±1 °C, and the light cycle was controlled as 12 h light and 12 h dark (lights on at 7 am). Mice were transferred from the animal housing facility to the phenotyping area at 10 weeks of age. On testing days, animals were transferred to the antechambers of the experimental room 30 min before the start of the experiment. All experiments were performed between 8:00 AM and 2:00 PM. A resting period of 2 days to 1 week was used between two consecutive tests. The body weights of animals were recorded once a week (on the same day and at the same time) from the age of 12 weeks until the day of euthanasia.

Animals at the same age passed through the same behavioural pipeline and data were pooled. Tests were administered in the following order: open field (13 weeks), novel object recognition with a 3 hour delay (14 weeks), three-chamber test (15 weeks), Morris water maze (16-17 weeks), social interaction (18 weeks), rotarod (19 weeks), grip test (19 weeks), and fear conditioning (20 weeks).

To study the behavior of *Kansl1*^+/-^ mice, we generated two cohorts. The first cohort comprised 8 *Kansl1^+/-^* and 10 wt animals. Tests were administered in the following order: circadian activity (13 weeks), elevated plus maze (14 weeks), open field (15 weeks), novel object recognition with a 3 hour delay (15 weeks), repetitive behaviors (16 weeks), rotarod (18 weeks), grip test (18 weeks), and fear conditioning (19 weeks). Body weights of animals were recorded at 15, 17 and 19 weeks of age. The second cohort comprised 9 *Kansl1^+/-^* and 12 wt mice and was devoted to the three-chamber test (15 weeks) and social interaction test (17 weeks).

Circadian activity (AC) was measured to assess spontaneous activity and feeding behaviour over the complete light/dark cycle. Testing was performed in individual cages (11 x 21 x 18 cm^3^) fitted with infrared captors linked to an electronic interface (Imetronic, France) that provided automated measures of position and locomotor activity. Mice were put into cages at 11 am on the first day and removed on the next day at 7 pm. The light cycle was controlled as 12 h light and 12 h dark (lights on at 7 am). The 32 hours of testing were divided into three different phases: the habituation phase (from 11 am to 7 pm on the first day); the night/dark phase (from 7 pm on the first day to 7 am on the second day); and the day/light phase (from 7 am to 7 pm on the second day). Feeding behavior was evaluated using an automated lickometer and a 20 mg pellet feeder (Test Diet, Hoffman La-Roche).

Open-field was used to evaluate exploration behaviour. Mice were tested in automated open fields (44.3 x 44.3 x 16.8 cm) made of PVC with transparent walls and a black floor, and covered with translucent PVC (Panlab, Barcelona, Spain). The open field arena was divided into central and peripheral regions and was homogeneously illuminated at 150 Lux. Each mouse was placed on the periphery of the open field and allowed to explore the apparatus freely for 30 min. The distance travelled, the number of rears and time spent in the central and peripheral parts of the arena were recorded over the test session.

Anxiety and activity of mice were evaluated with the elevated plus maze. The apparatus consists of two opposed open arms (30 x 5 cm) crossed by two enclosed arms (30 x 5 x 15 cm), and elevated 66 cm from the floor. The light intensity at the extremity of the open arms was kept at 50 Lux. Each mouse was tested for 5 min after being placed in the central platform and allowed to explore freely the apparatus. The number of entries and time spent in the open arms were used as an anxiety index. Closed arm entries and rears in the closed arms were used as measures of general motor activity.

For studying repetitive behaviour, mice were put individually into dimly lit (60 Lux) clean home-cages without pellets or water bottle. The occurrence of repetitive behaviours (rearing, jumping, climbing, digging, grooming) was observed for 10 min and scored using an ethological keyboard (Viewpoint, Labwatcher, France).

Sensorimotor coordination and balance were assessed with the rotarod test. The apparatus (Biosed, France) is a rotating bar of 5 cm diameter (hard plastic materiel covered by grey rubber foam) on which mice are placed facing the direction of rotation. Animals were ﬁrst habituated to stay on the rod for 30 seconds at a constant speed of 4 rotations per minute (rpm). This was followed by 3 training days with 4 trials per day. Mice were placed on an accelerating rod increasing from 4 rpm to 40 rpm in a 5 min period. The test was stopped when mice fell down from the rod or when they made more than one passive rotation. The latency to fall and the maximal speed before falling was recorded.

The muscular strength was evaluated with the grip strength. Mice were first weighed and tested with a handy force gauge (Bioseb, France). Animals were placed on the instrument grid and pulled by the tail until letting go. The force (g) was related to animal weight (g).

The Y-maze test was used to evaluate short-term working memory. This test is based on the innate preference of animals to explore an arm that has not been previously explored, a behavior that, if occurring with a frequency greater than 50%, is called spontaneous alternation. In this test, we used a Y-shaped maze with three white, opaque plexiglass arms of equivalent length forming a 120° angle with each other. The arms have walls with specific motifs to distinguish from each other. After introduction at the center of the maze illuminated at 60 Lux, animals were allowed to explore freely the three arms for 6 minutes. The number of arm entries and the number of triads were recorded to calculate the percentage of alternation.

The novel object recognition (NOR) memory is based on the innate preference of rodents to explore novelty. On the first day, mice were habituated to the arena for 30 min at 60 Lux. On the following day, animals were submitted to the first 10-min acquisition trial during which they were individually placed in the presence of object A (marble or die) placed 10 cm away from one of the box corners. The exploration time of object A (when the animal’s snout was directed towards the object at a distance ≤1 cm) was recorded. A 10-min retention trial (choice trial) was conducted 30 min, 3 h or 24 h later. The familiar object (object A) and the novel object (object B) were placed at a distance 10 cm from two open field corners (the distance between the two objects was approximately 20 cm) and the exploration time of these two objects was recorded. A discrimination index was defined as (*t*_B_/(*t*_A_ + *t*_B_)) × 100. All mice that did not explore the first object for more than 3 seconds during the acquisition trial were excluded from the analysis.

The Morris water maze paradigm was used to evaluate spatial learning and memory of mice. The protocol is adapted from the already established protocol [4]. The apparatus consists in a circular pool (150-cm diameter, 60-cm height) filled to a depth of 40 cm with water maintained at 20°C–22°C and made opaque using a white aqueous emulsion (Acusol OP 301 opacifier). An escape platform, made of 6 cm diameter rough plastic, is submerged 1 cm below the water surface. The test began with 6 days of acquisition, 4 trials per day, at 120 Lux. Each trial started with the mice facing the interior wall of the pool and ended when animals climb on the platform or after a maximum searching time of 90 sec. The platform was at the same position for all the four trials but starting positions changed randomly between each trial with departures from each cardinal point. Travelled distances to find the platform and swimming speeds were analyzed each day. On 7^th^ day, mice were given a single trial of 60 seconds trial during the probe test or removal session in which the platform has been removed. The distance traveled and duration spent in each quadrant (NW, NE, SW, SE) were recorded. Annulus crossing index was calculated as the number of times that animals crossed the exact platform position. On 12^th^ and 13^th^ days, mice were given reversal sessions with 4 trials of 90 seconds per day. The platform was made visible by a small dark ball placed 12cm on top of the platform, while the external cues were hidden by surrounding the pool with a black curtain. In order to be sure that the mouse used the platform cue, starting position and platform position were changed for each trial.

The fear conditioning test was used to assess aversive learning and memory. Polymodal operant chambers (Coulbourn Instruments, USA) consisting of steel rod floor and plexiglas walls of 18.5 x 18 x 21.5 cm are used for this test. On the first day, during the conditioning session, mice were allowed to acclimate in the chambers for 4 min, then a light/tone (10 kHz, 80-dB) conditioned stimulus (CS) was presented for 20 sec and coterminated by a mild (0.4 mA, 1 sec) footshock unconditioned stimulus (US). Mice were left in the chambers for another 2 min then were put back to their homecages. Context session was performed 24 hours after the conditioning session. Mice were placed into the same chambers and allowed to explore for 6 min without presentation of light/auditory CS. Cue session was performed 5 hours after the context session. Animals were tested in distinct appearance chamber (grey plastic ground, black opaque plastic walls, change of light orientation) and were allowed to habituate for 2 min then presented to light/auditory CS. This sequence was repeated once again. During each session, animal movement was monitored to detect freezing behavior.

Cued fear extinction was tested in the various genotypes accordingly to previous report[5]. Briefly, mice were housed individually in a ventilation area before the start of behavioral training. To avoid for high stress during experiments, animals were handled every day before the start of the experiment during a week. On day 1, animals were transferred to the conditioning context (context A) for habituation. Both CS+ (total CS duration of 30s, consisting of 50-ms pips repeated at 0.9 Hz, pip frequency 7.5 kHz, 80 dB sound pressure level) and CS- (30s, consisting of white noise pips repeated at 0.9Hz, 80dB sound pressure level) were presented 4 times with a variable inter stimulus interval (ISI). On day 2 we proceeded with the conditioning phase. The protocol consisted of 5 pairings of CS+ with the US onset coinciding with the CS+ offset (1s foot shock, 0.6mA, ISI 10-60s). CS- presentations were intermingled with CS+ presentations and ISI was variable over the whole training course. Cued memory was tested 24 hours after conditioning by analyzing the freezing levels at the first CS+ presentations in context B (recall). Fear extinction was induced by submitting mice to 24 CS+-alone in two sessions (days 3 and 4). The within session extinction was evaluated by comparing freezing levels at CS+A remote cued fear test (retrieval) was performed 7 days after the extinction sessions. Freezing behavior was quantified automatically in each session using a fire-wire CCD-camera connected to automated freezing detection software (Ugo Basile, Italy).

Social behaviour was tested using the three-chamber sociability test for social preference and discrimination using a specific apparatus (Stoelting, Dublin) with three successive and identical chambers (20 cm × 40 cm × 22 (height) cm with 5 cm × 8 cm openings allowing access between the chambers). During habituation, mice were allowed to explore the three chambers freely for 10 min. In the second phase (social interest session), the test mouse was placed into the central box, while an unfamiliar mouse (stranger 1) was put into one of the restrictive areas (wire cages) in a random and balanced manner. The doors were reopened and the test mouse was allowed to explore the chambers for 10 min. Time spent to explore the empty cage and the cage with stranger 1 was recorded. In the third phase (social discrimination session), the social discrimination was evaluated with a new mouse being placed into the empty wire cage and the test mouse was allowed to explore again the entire arena for 10 min, having the choice between the familiar mouse (stranger 1) and the novel mouse (stranger 2). A delay of 2 minutes (necessary to put stranger 1 or 2 in the restrictive areas) was used between each session. Animal used as strangers were of similar sex (males) and genotype (C57BL/6N) than experimental animals but were of younger age (10 weeks versus 15weeks for experimental animals) in order to reduce aggressiveness. A social discrimination index or social preference was defined as the percentage of time spent exploring the novel mouse as (*t*_2_/(*t*_1_ + *t*_2_)) × 100.

In addition to three-chamber sociability test, social behaviour was tested using the social interaction test. In each session, 2 mice of the same genotype and similar body weight housed in different cages were put in an open field area for 10 min. The duration of sniffing and social behaviours were recorded during the 10-min session.

Body composition and body size analysis were performed on 20-week old animals. Fat content was examined by using a nuclear magnetic resonance apparatus and Minispec+ analyser (Bruker, Germany). The test was conducted during the light period on conscious fed mice. For body size analysis, animals were anesthetized under isoflurane and distances from snout to tail were measured with a FST ruler (Fine Science Tools).

## Craniofacial analysis

Craniums of 13 week-old female mice (*n* = 10 wt and 10 *Del/+; n* = 9 wt and 9 *Dup/+*) were stored in 100% ethanol. Three-dimensional coordinates of 39 relevant cranial landmarks were recorded using Landmark software, and posterior comparisons were performed using the Euclidean distance matrix analysis (EDMA) with the WinEDMA software (version 1.0.1 beta). Three-dimensional data were converted into linear distances compiled into a matrix. Both the form (size of the skull) difference matrix (FDM) and the shape difference matrix (SDM) were analysed. A ratio different from 1 (FDM) or 0 (SDM) for any linear distance indicates that the two samples are not similar for that measure.

Confidence intervals were estimated using a non-parametric bootstrapping algorithm. For each linear distance, the null hypothesis was rejected if the 90% confidence interval did not include 1 (FDM) or 0 (SDM): rejection of the null hypothesis enabled localization of differences to specific landmarks and linear distances. Bootstrap Distributions of T (FDM) and Z (SDM) were calculated with the corresponding value for the samples. The principal component analysis was carried out using the MorphoJ analysis tool[6].

## MRI processing and Analysis

A multi-channel 7.0 T, 40 cm diameter bore magnet (Varian Inc. Palo Alto, CA) was used to acquire anatomical images. A custom-built 16-coil solenoid array was used to image 16 samples concurrently[7] Parameters used in the scans were optimized for gray–white matter contrast: a T2-weighted 3D fast spin-echo sequence, with TR = 2000 ms, echo train length = 6, TEeff = 42 ms, field-of-view (FOV) = 25 × 28 × 14 mm and matrix size = 450 × 504 × 250, giving an image with 56-micron isotropic voxels and an optimal signal to noise ratio (SNR) in the order of 40[8]. Total imaging time was 11.7 h. Scans were corrected for geometric distortions generated by the image acquisition process based on images of precision machined phantoms. Four of the samples in this study were excluded from the analysis: two samples showed hydrocephalus in the ventricles (both *Dup/+*), one sample contained a large mass in the middle of the brain (wt), and one sample failed in the image registration (*Del/+*), resulting in a cohort consisting of 8 *Del/+*, 10 wt, 11 *Del/Dup*, and 8 *Dup/+* mice.

The MRI scans were linearly (6 parameters followed by 12 parameters) and subsequently non-linearly registered. All scans were then resampled with the appropriate transform and averaged to create a population atlas, which represents the average anatomy of all brains. Registrations were performed with a combination of the mni_autoreg tools[9] and ANTs (Advanced Normalization Tools)[10, 11]. The result of this registration is to have all scans deformed into exact alignment with each other in an unbiased fashion. This allows for the analysis of the deformations needed to take each individual brain into the final atlas space, the goal being to model how the deformation fields relate to genotype[12]. The Jacobian determinants of the deformation fields are then used to estimate the volume changes in each voxel. Significant regional volume changes can then be calculated in two different ways. First, regional measurements can be calculated by registering a pre-existing classified MRI atlas on to the population atlas, which allows for the volume measurement of 159 different brain regions[13-15]. Second, individual voxel measurements can be calculated from comparisons of the Jacobian determinants in a specific voxel between the *Del/+*, *Del/Dup*, *Dup/+* and wt mice. All statistical analyses were performed in the R statistical environment (www.r-project.org). Multiple comparisons were controlled for by using the false discovery rate (FDR) [16]. We used a very stringent 0.1% FDR threshold to determine significance.

## Hippocampal slice electrophysiology

Acute hippocampal slices were used to record field excitatory post synaptic potentials (fEPSPs), by the MEA60 electrophysiological suite (Multi Channel Systems, Reutlingen, FRG) as described [17, 18]. Eight set-ups consisting of a MEA1060-BC pre-amplifier and a filter amplifier (gain 550x) were run simultaneously by data acquisition units operated by MC_Rack software. Raw electrode data were digitized at 10 kHz and stored on a PC hard disk for subsequent analysis. To record fEPSPs, a hippocampal slice was placed into the well of the 5x13 3D multi electrode array (MEA) biochip (Qwane Biosciences, Lausanne, Switzerland). The slice was guided to a desired position with a fine paint brush and gently fixed over MEA electrodes by a silver ring with attached nylon mesh lowered vertically by a one-dimensional U-1C micromanipulator (You Ltd, Tokyo, Japan). MEA biochips were fitted into the pre-amplifier case and fresh ACSF was delivered to the MEA well through a temperature-controlled perfusion cannula that warmed perfused media to 32°C. Monopolar stimulation of Schäffer collateral /commissural fibers through array electrodes was performed by STG4008 stimulus generator (Multi Channel Systems, Reutlingen, FRG). Biphasic (positive/negative, 100 µs/a phase) voltage pulses were used. Amplitude, duration and frequency of stimulation were controlled by MC_Stimulus II software. All experiments were performed using two-pathway stimulation of Schäffer collateral/commissural fibers. Our previous experiments that utilized MEAs, demonstrated that largest LTP was recorded in proximal part of apical dendrites of CA1 pyramidal neurons [17]. We have therefore picked a single principal recording electrode in the middle of the proximal part of the CA1 region and assigned two electrodes for stimulation of the control and test pathways on the subicular side and on the CA3 side of *stratum radiatum* respectively. The distance from the recording electrode to the test stimulation electrode was 400-510 µm and to the control stimulation electrode 316-447 µm. To evoke orthodromic fEPSPs, test and control pathways were activated in succession at a frequency of 0.02 Hz. Baseline stimulation strength was adjusted to evoke a response that corresponded to 40% of the maximal attainable fEPSP at the recording electrode located in proximal *stratum radiatum*. Slope of the negative part of fEPSPs was used as a measure of the synaptic strength. Paired stimulation with an interpulse interval of 50 ms was used to observe paired-pulse facilitation (PPF) in baseline conditions in the test pathway before LTP induction. PPF was calculated by dividing the negative slope of fEPSP obtained in response to the second pulse by the amplitude of fEPSP amplitude evoked by the preceding pulse. To induce LTP, 10 bursts of baseline strength stimuli were administered at 5 Hz to test pathway with 4 pulses given at 100 Hz per burst (total 40 stimuli). LTP plots were scaled to the average of the first five baseline points. Normalization of LTP values was performed by dividing the fEPSP amplitude in the tetanized pathway by the amplitude of the control fEPSP at corresponding time points. Normalized LTP values averaged across the period of 61-65 min after theta-burst stimulation were used for statistical comparison.

In electrophysiological experiments, since several slices were routinely recorded from every mouse, fEPSPmax slopes, PPF and LTP values between wt and mutant mice were compared using two-way nested ANOVA design with genotype (group) and mice (sub-group) as fixed and random factors respectively (STATISTICA v.10, StatSoft, USA). DF error was computed using the Satterthwaite’s method and main genotype effect was considered significant if *P* < 0.05. Graph plots and normalization were performed using OriginPro 8.5 (OriginLab, Northampton, USA). Electrophysiological data are presented as the mean ± s.e.m. with *n* and *N* indicating number of slices and mice respectively.

## S1 appendix references

1. Michel D, Chatelain G, Herault Y, Harper F, Brun G. H-DNA can act as a transcriptional insulator. Cell Mol Biol Res. 1993;39(2):131-40. PubMed PMID: 8220583.

2. Brault V, Pereira P, Duchon A, Herault Y. Modeling chromosomes in mouse to explore the function of genes, genomic disorders, and chromosomal organization. Plos Genetics. 2006;2(7):911-9. doi: e86

10.1371/journal.pgen.0020086. PubMed PMID: WOS:000239494800001.

3. Herault Y, Chatelain G, Brun G, Michel D. The PUR element stimulates transcription and is a target for single strand-specific binding factors conserved among vertebrate classes. Cell Mol Biol Res. 1993;39(8):717-25. PubMed PMID: 7951411.

4. Duchon A, Pothion S, Brault V, Sharp AJ, Tybulewicz VLJ, Fisher EMC, et al. The telomeric part of the human chromosome 21 from Cstb to Prmt2 is not necessary for the locomotor and short-term memory deficits observed in the Tc1 mouse model of Down syndrome. Behavioural Brain Research. 2011;217(2):271-81. doi: 10.1016/j.bbr.2010.10.023. PubMed PMID: WOS:000286698300003.

5. Houbaert X, Zhang CL, Gambino F, Lepleux M, Deshors M, Normand E, et al. Target-specific vulnerability of excitatory synapses leads to deficits in associative memory in a model of intellectual disorder. J Neurosci. 2013;33(34):13805-19. doi: 10.1523/JNEUROSCI.1457-13.2013. PubMed PMID: 23966701.

6. Klingenberg CP. MorphoJ: an integrated software package for geometric morphometrics. Mol Ecol Resour. 2011;11(2):353-7. doi: 10.1111/j.1755-0998.2010.02924.x. PubMed PMID: 21429143.

7. Lerch JP, Yiu AP, Martinez-Canabal A, Pekar T, Bohbot VD, Frankland PW, et al. Maze training in mice induces MRI-detectable brain shape changes specific to the type of learning. Neuroimage. 2011;54(3):2086-95. doi: 10.1016/j.neuroimage.2010.09.086. PubMed PMID: 20932918.

8. Kale SC, Lerch JP, Henkelman RM, Chen XJ. Optimization of the SNR-resolution tradeoff for registration of magnetic resonance images. Hum Brain Mapp. 2008;29(10):1147-58. doi: 10.1002/hbm.20453. PubMed PMID: 17957707.

9. Collins DL, Neelin P, Peters TM, Evans AC. Automatic 3D intersubject registration of MR volumetric data in standardized Talairach space. J Comput Assist Tomogr. 1994;18(2):192-205. PubMed PMID: 8126267.

10. Avants BB, Tustison NJ, Song G, Cook PA, Klein A, Gee JC. A reproducible evaluation of ANTs similarity metric performance in brain image registration. Neuroimage. 2011;54(3):2033-44. doi: 10.1016/j.neuroimage.2010.09.025. PubMed PMID: 20851191; PubMed Central PMCID: PMCPMC3065962.

11. Avants BB, Epstein CL, Grossman M, Gee JC. Symmetric diffeomorphic image registration with cross-correlation: evaluating automated labeling of elderly and neurodegenerative brain. Med Image Anal. 2008;12(1):26-41. doi: 10.1016/j.media.2007.06.004. PubMed PMID: 17659998; PubMed Central PMCID: PMCPMC2276735.

12. Nieman BJ, Flenniken AM, Adamson SL, Henkelman RM, Sled JG. Anatomical phenotyping in the brain and skull of a mutant mouse by magnetic resonance imaging and computed tomography. Physiol Genomics. 2006;24(2):154-62. doi: 10.1152/physiolgenomics.00217.2005. PubMed PMID: 16410543.

13. Dorr AE, Lerch JP, Spring S, Kabani N, Henkelman RM. High resolution three-dimensional brain atlas using an average magnetic resonance image of 40 adult C57Bl/6J mice. Neuroimage. 2008;42(1):60-9. doi: 10.1016/j.neuroimage.2008.03.037. PubMed PMID: 18502665.

14. Ullmann JF, Watson C, Janke AL, Kurniawan ND, Reutens DC. A segmentation protocol and MRI atlas of the C57BL/6J mouse neocortex. Neuroimage. 2013;78:196-203. doi: 10.1016/j.neuroimage.2013.04.008. PubMed PMID: 23587687.

15. Steadman PE, Ellegood J, Szulc KU, Turnbull DH, Joyner AL, Henkelman RM, et al. Genetic effects on cerebellar structure across mouse models of autism using a magnetic resonance imaging atlas. Autism Res. 2014;7(1):124-37. doi: 10.1002/aur.1344. PubMed PMID: 24151012; PubMed Central PMCID: PMCPMC4418792.

16. Genovese CR, Lazar NA, Nichols T. Thresholding of statistical maps in functional neuroimaging using the false discovery rate. Neuroimage. 2002;15(4):870-8. doi: 10.1006/nimg.2001.1037. PubMed PMID: 11906227.

17. Hunter CL, Isacson O, Nelson M, Bimonte-Nelson H, Seo H, Lin L, et al. Regional alterations in amyloid precursor protein and nerve growth factor across age in a mouse model of Down's syndrome. Neurosci Res. 2003;45(4):437-45. PubMed PMID: 12657457.

18. Hyde LA, Crnic LS. Age-related deficits in context discrimination learning in Ts65Dn mice that model Down syndrome and Alzheimer's disease. Behav Neurosci. 2001;115(6):1239-46. PubMed PMID: 11770055.
