# Supplementary figures and images for: Mouse models of 17q21.31 microdeletion and microduplication syndromes highlight the importance of Kansl1 for cognition
Source: PLoS Genet. 2017 Jul 13;13(7):e1006886. doi: 10.1371/journal.pgen.1006886 (PMC5531616; doi:10.1371/journal.pgen.1006886)

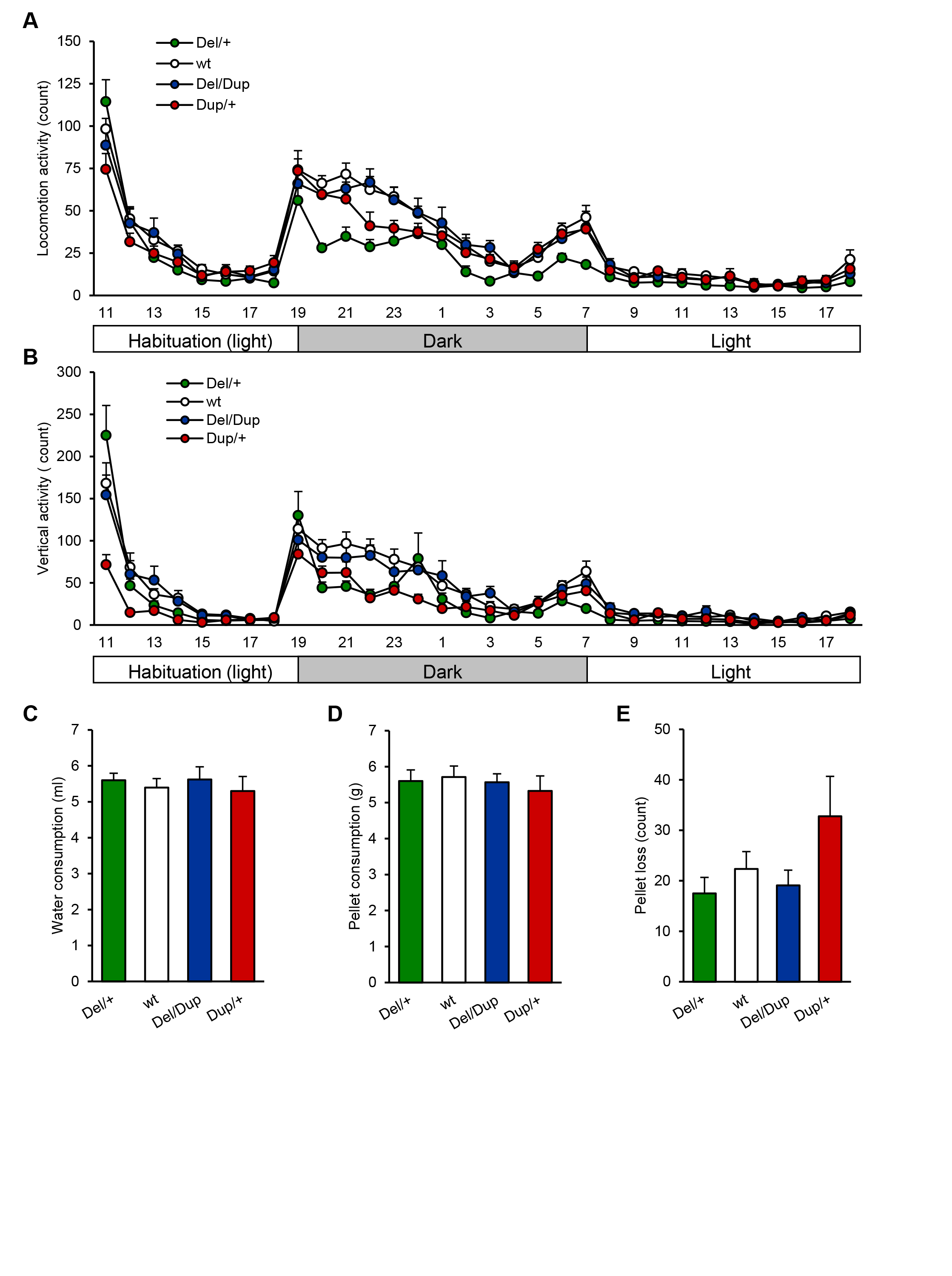

Supplement: S1 Fig — Patterns of locomotor activity (A) and vertical activity (B) during the 32-hours of test. (C-D) Feeding behaviors. Water (C) and pellet (D) consumption during the 32-hours of testing. (E) Pellets lost by animals which passed through the holed ground. Del/+, Dup/+, and Del/Dup animals showed normal pattern of activity and consumption during the test. Data are represented as the mean + s.e.m.. (TIF) [file pgen.1006886.s002.tif]

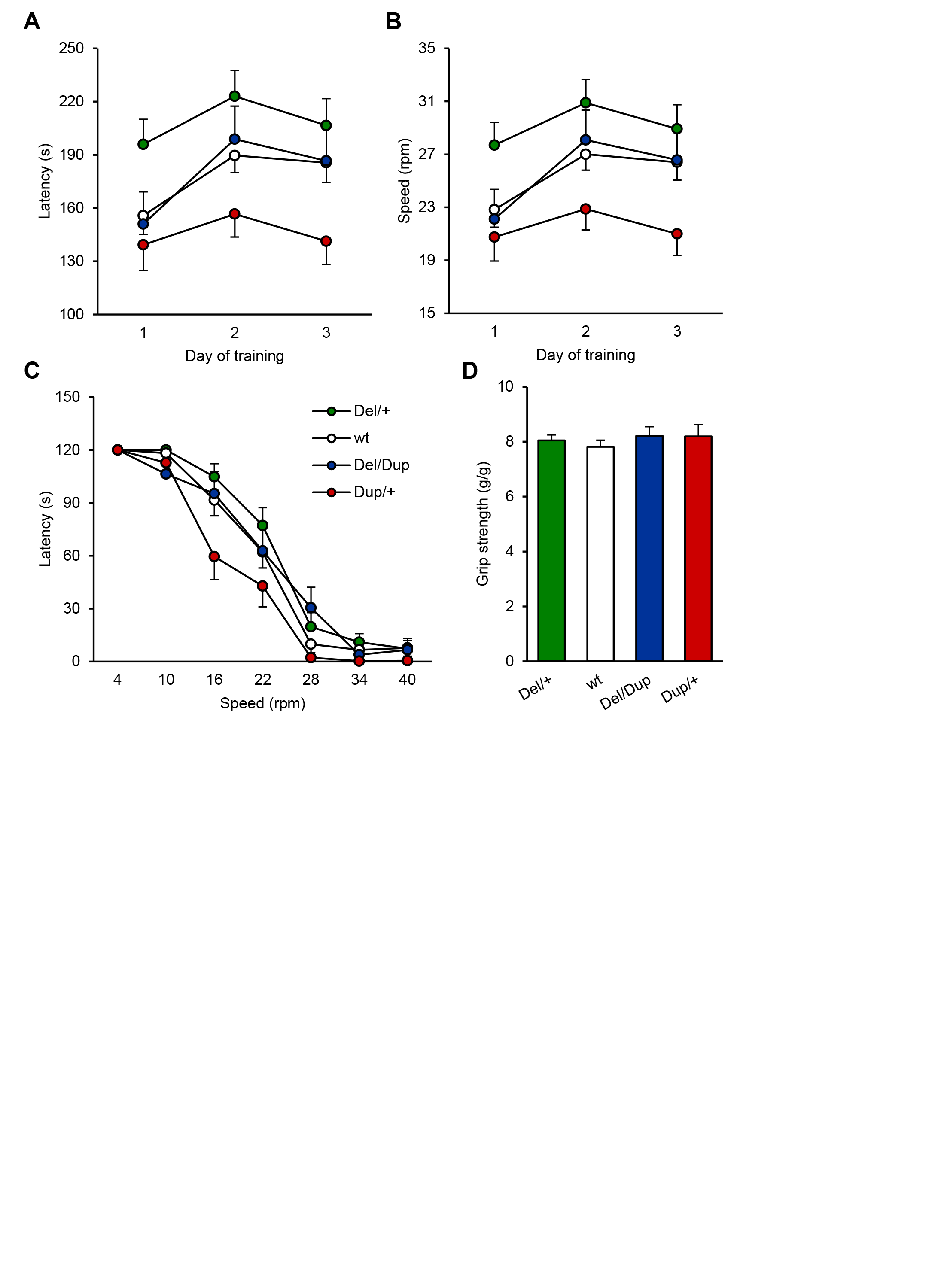

Supplement: S2 Fig — (A-B) Training phase of the rotarod test. (A) Results are expressed as the time (s) that mice remained on an accelerating rod (4–40 rpm over 5 min) before falling. (B) Corresponding rotational velocity (rpm) at the time of falling. (C) Challenge phase of the rotarod test. The graph plots the time (s) that mice stayed on the rod when tested at constant speeds between 4 and 40 rpm. (D) Four-paw grip test. We did not detect locomotor coordination or muscular defects in the mutant mice. Graphs depict mean + s.e.m.. (TIF) [file pgen.1006886.s003.tif]

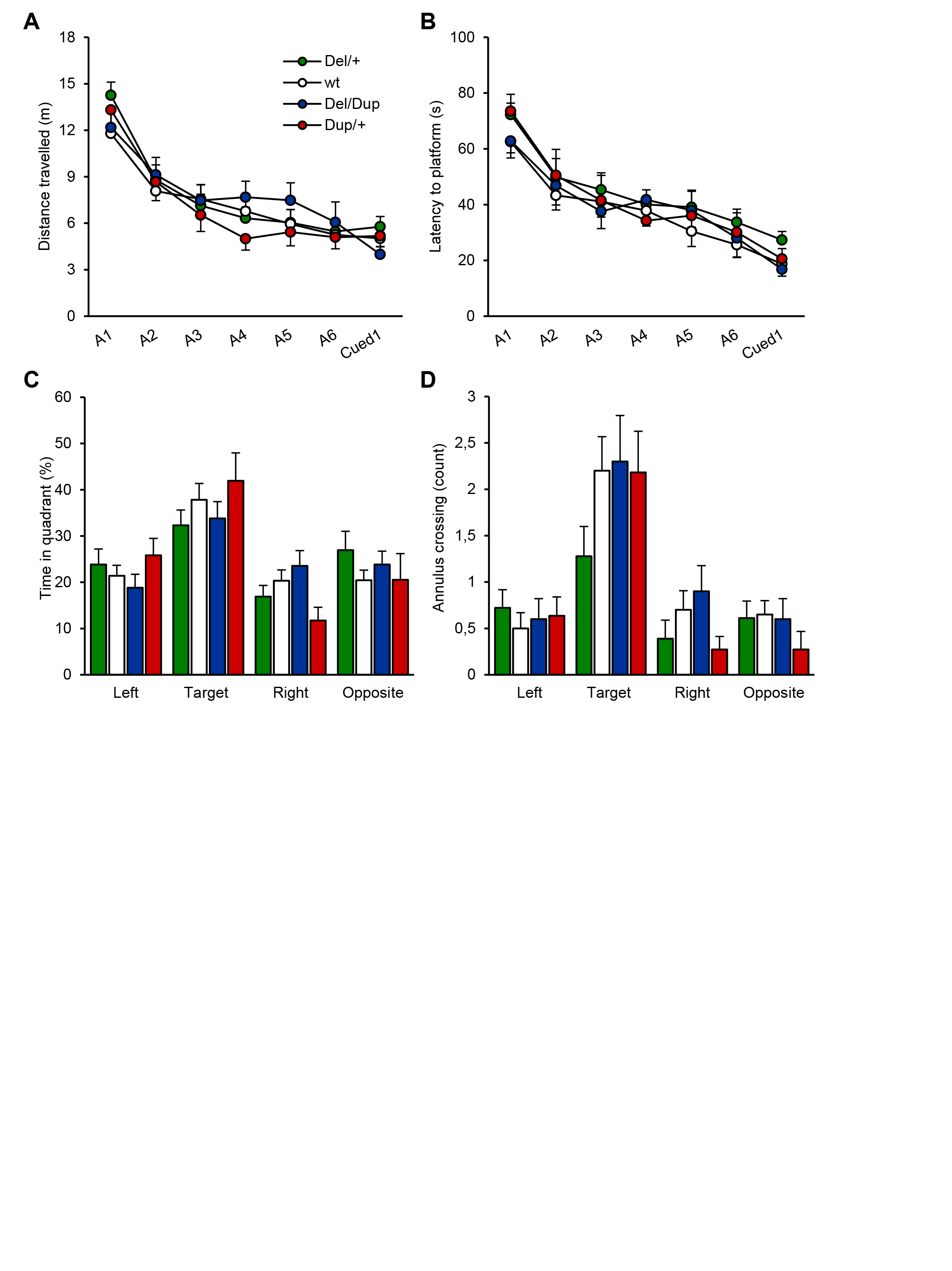

Supplement: S3 Fig — (A-B) Acquisition phase. (A) Distance travelled (m) to find the platform along acquisition (A1 –A6) and reversal (Cued1) sessions. (B) Corresponding latency (s) to find the platform. (C-D) Removal phase. Mice were scored for the percentage of time spent in the different quadrant of the MWM (C) and the annulus crossing counts (D) during one single trial where the platform was removed. Del/+, Dup/+, and Del/Dup animals do not show any learning and memory defects in the MWM test. All graphs depict mean + s.e.m.. (TIF) [file pgen.1006886.s004.tif]

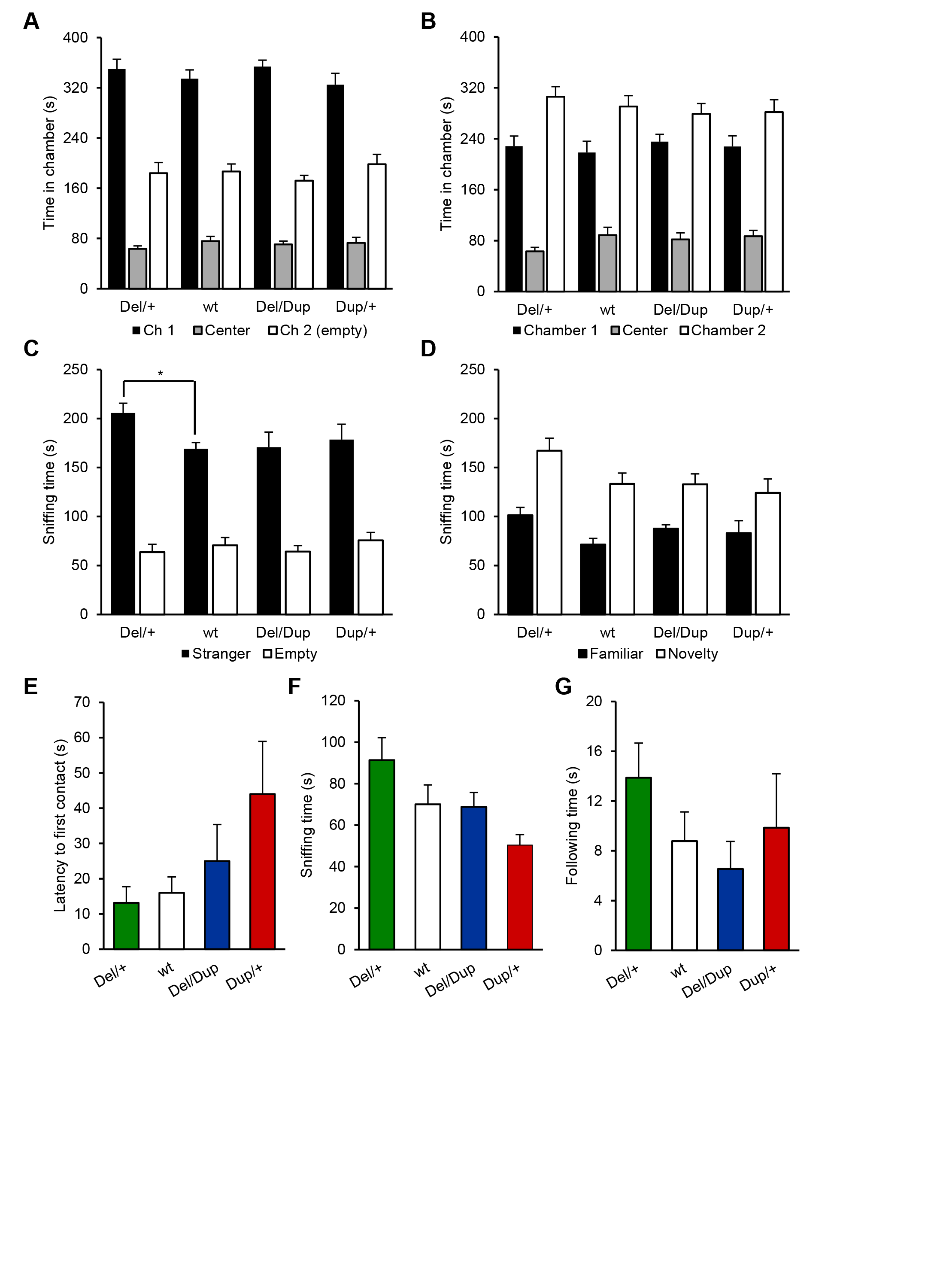

Supplement: S4 Fig — (A, C) Social interest session. (B, D) Social discrimination session. (A-B) Time (s) in chambers during the social interest session (A) in which restrictive area of chamber 1 contains an unfamiliar mouse and during the social discrimination session (B) where chamber 1 and 2 restrictive areas contain respectively familiar and unfamiliar mice. (C-D) Corresponding time (s) spent to explore restrictive areas. Compared to wt littermates, Del/+ animals spent more time to interact with the stranger mouse during the social interest session. All genotypes show similar social discrimination capacities. (E-G) Social interaction test. Latency to first contact (E), sniffing time (F) and following time (G) (s) of 2 animals of similar genotype from different house cages putting together in an open-field during 10 min. All graphs depict mean + s.e.m.. Tukey's test following a significant one-way ANOVA. *P < 0.05 vs wt. (TIF) [file pgen.1006886.s005.tif]

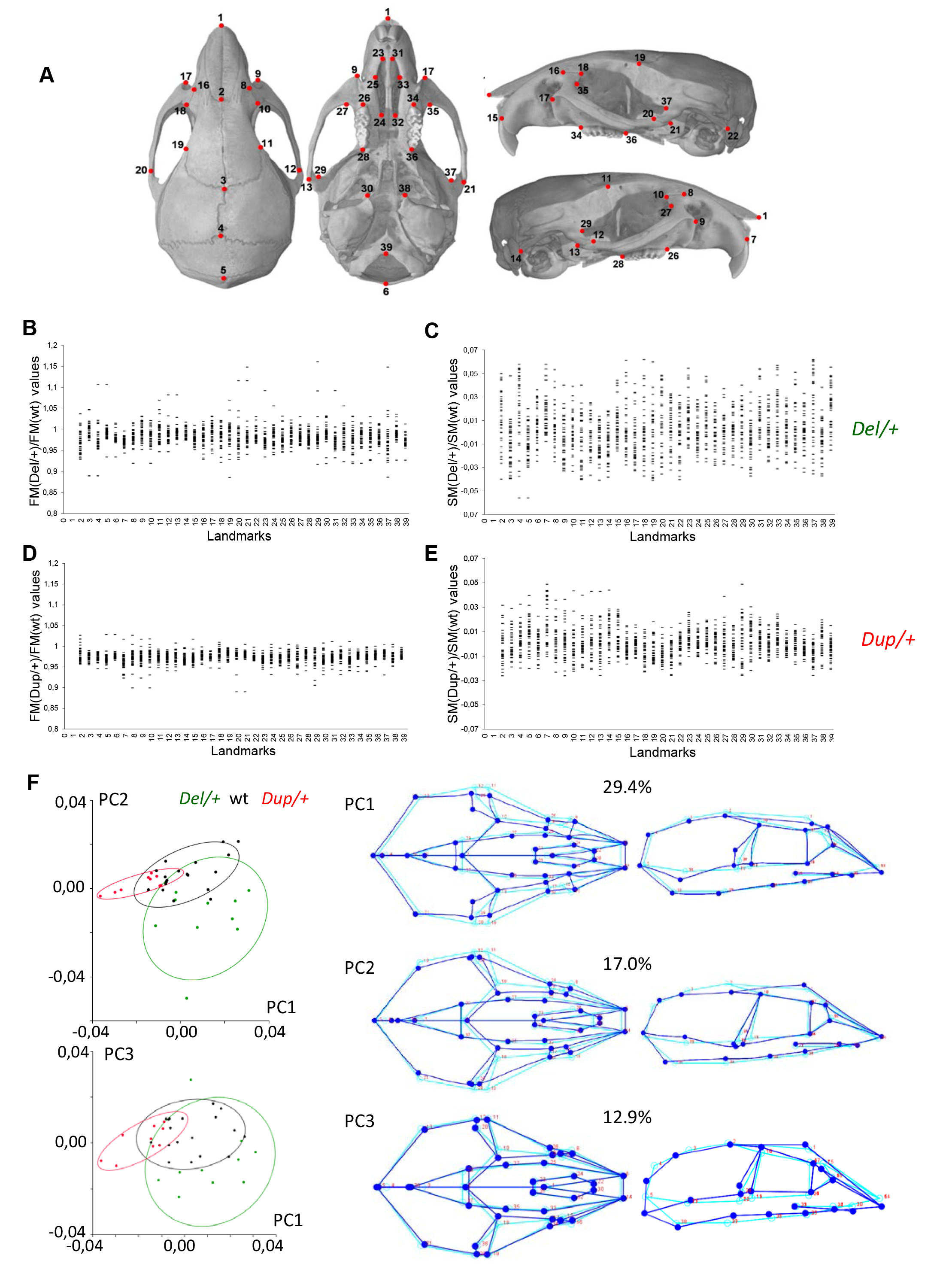

Supplement: S5 Fig — (A) Representative reconstructed 3D skull images with localisation of the 39 landmarks used in the study. Euclidian distances between the different landmarks allowed calculation of both the form (or size) difference (FD) and the shape difference (SD). Analysis revealed no defect of skull size in Del/+ (B) and Dup/+ (D) animals. Trends for skull shape alteration of Del/+ (C) and Dup/+ (E) mice were noticed. (F) Principal component analysis based on the 39 landmarks and symmetric component captured in the study using the MorphoJ software. The analysis allowed to segregate the 3 genotypes based on 3 main component PC1, PC2 and PC3 that affect the skull shape as showed by wireframe graphs with the shape of wt control in light blue and the shape associated with the component in dark blue. Landmarks are showed in red. (TIF) [file pgen.1006886.s006.tif]

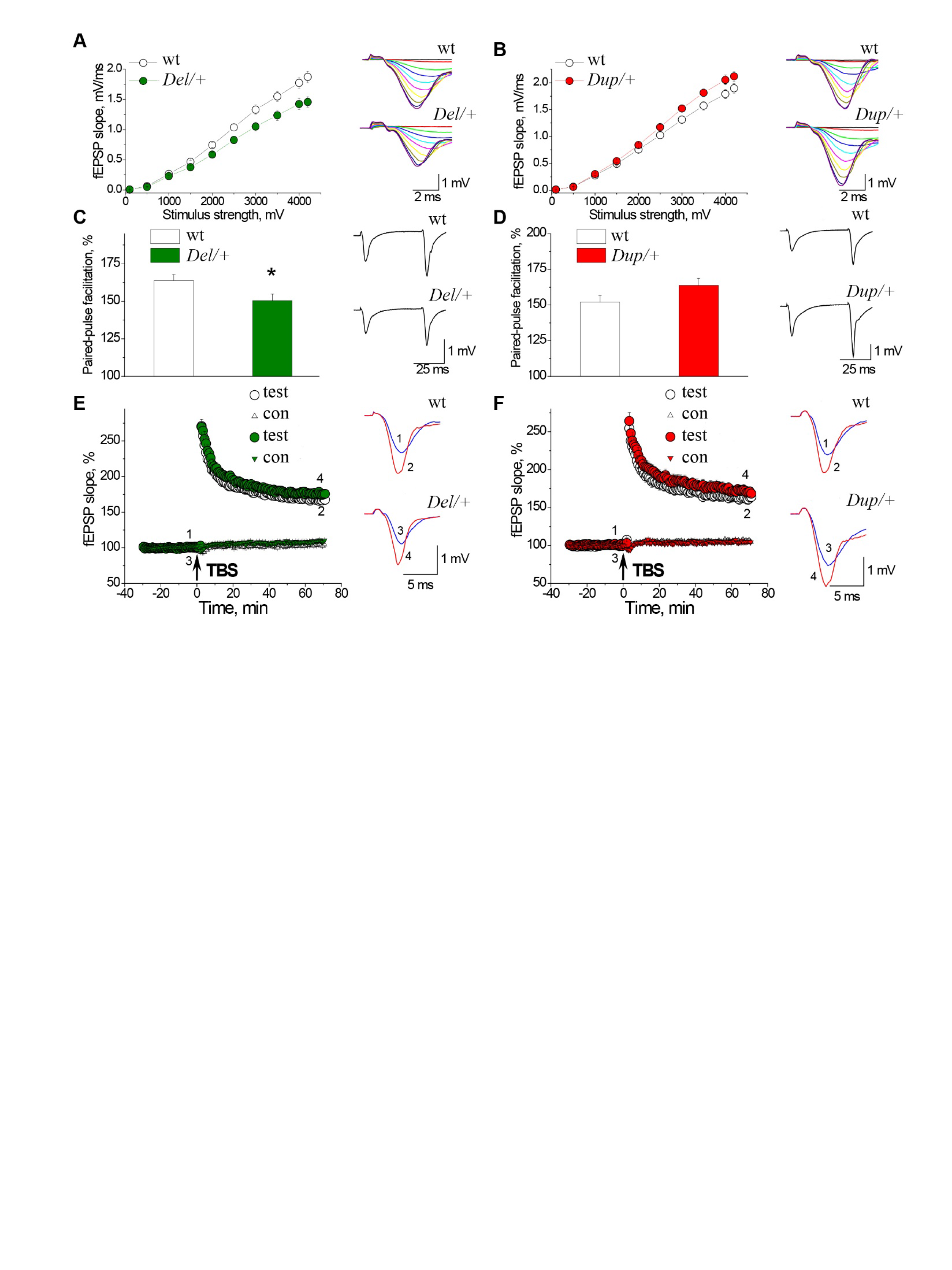

Supplement: S6 Fig — (A–B) Basal synaptic transmission. (A) Input-output relationships illustrate averaged fEPSP slopes in slices from Del/+ (n = 28; N = 9) and wt mice (n = 33; N = 8) in response to stimulation of Schäffer collaterals by biphasic voltage pulses of 0.1–4.2 V. Representative families of fEPSP traces are given on the right side. Synaptic responses were significantly lower in Del/+ mice (F(9, 531) = 7.59; P < 0.0001). Mean slopes of fEPSPs invoked by the maximum stimulus strength (4.2 V) were significantly smaller in slices from Del/+ mice (1.46 ± 0.09 mV/ms) than wt littermates (1.87 ± 0.09 mV; F(1,13.34) = 8.31; P = 0.025; two-way nested ANOVA, genotype effect). (B) Input-output relationships illustrate averaged fEPSP slopes in slices from Dup/+ (n = 27; N = 9) and wt mice (n = 27; N = 10). Basal synaptic transmission was nominally enhanced in Dup/+ mice: fEPSPmax mean slope was nominally higher in slices from Dup/+ mice (2.12 ± 0.09 mV/ms) than in slices from wt littermates (1.89 ± 0.09 mV/ms) but, the effect was not statistically significant (F(1,8.67) = 3.09; P = 0.114; two-way nested ANOVA, genotype effect). (C–D) Paired-pulse facilitation. (C) Paired-pulse facilitation was slightly but significantly lower in Del/+ animals (n = 28; N = 9) than in wt littermates (n = 33; N = 8; F(1, 11.04) = 6.506; P = 0.027). Representative fEPSP sweeps are presented on the right side. (D) Paired-pulse facilitation was nominally higher in Dup/+ animals (n = 27; N = 9) than wt littermates (n = 27; N = 10), but the effect did not reach statistical significance (F(1, 4.92) = 5.402; P = 0.069). (E–F) Theta-burst stimulation elicited pathway-specific long-term potentiation (LTP) of synaptic transmission in hippocampal CA1 area. (E) Normalized magnitude of LTP 60–65 min after LTP induction was similar in Del/+ mice (163 ± 4%; n = 27; N = 9; P = 0.88) and in their wt counterparts (165 ± 8%; n = 33; N = 8). Examples of test pathway fEPSP traces immediately before and 1 h after theta-burst st [file pgen.1006886.s007.tif]

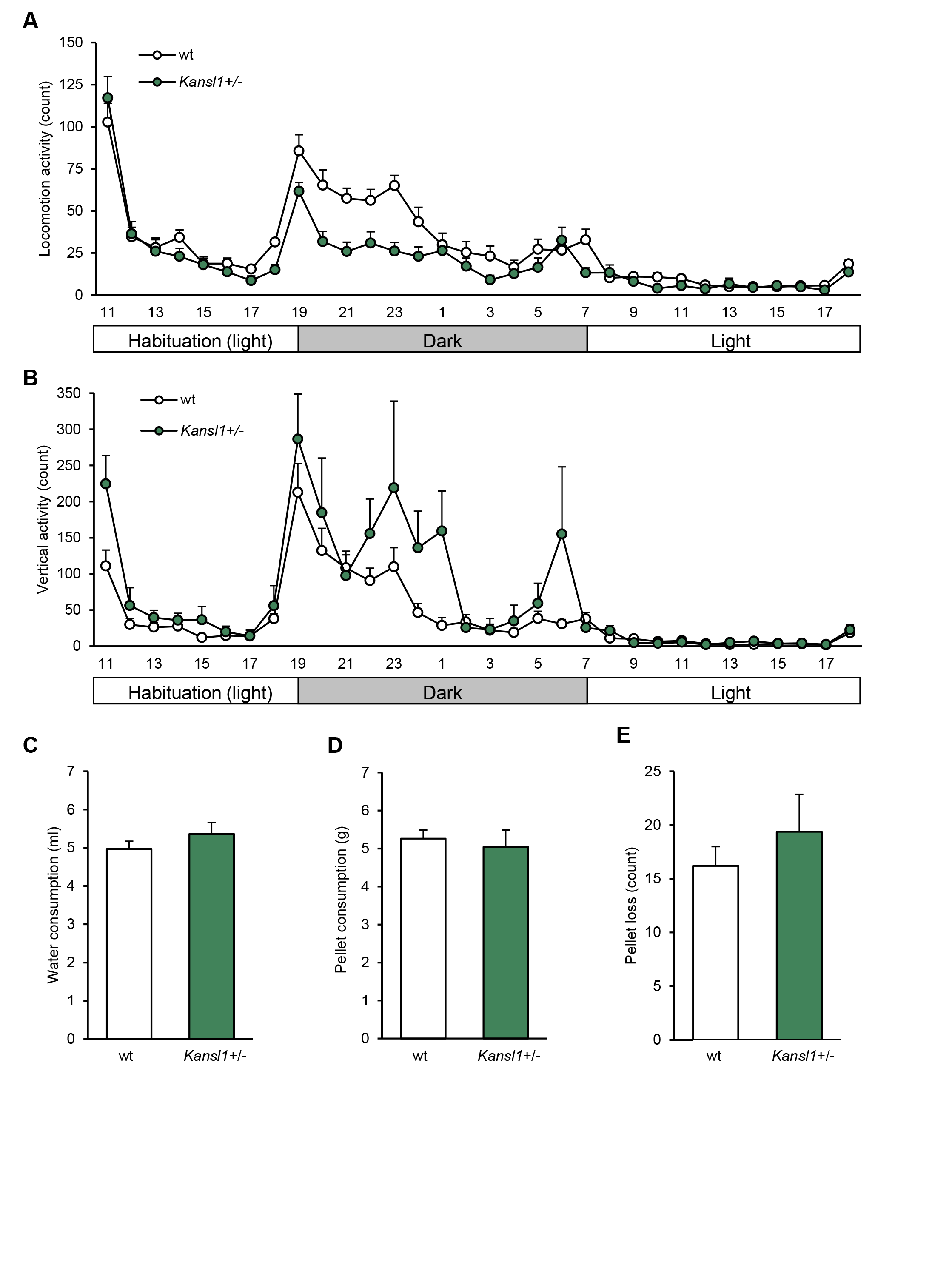

Supplement: S7 Fig — Patterns of locomotor activity (A) and vertical activity (B) during the 32-hours of test. (C-D) Feeding behaviors. Water (C) and pellet (D) consumption during the 32-hours of testing. (E) Pellets lost by animals which passed through the holed ground. Data are represented as the mean + s.e.m.. (TIF) [file pgen.1006886.s008.tif]

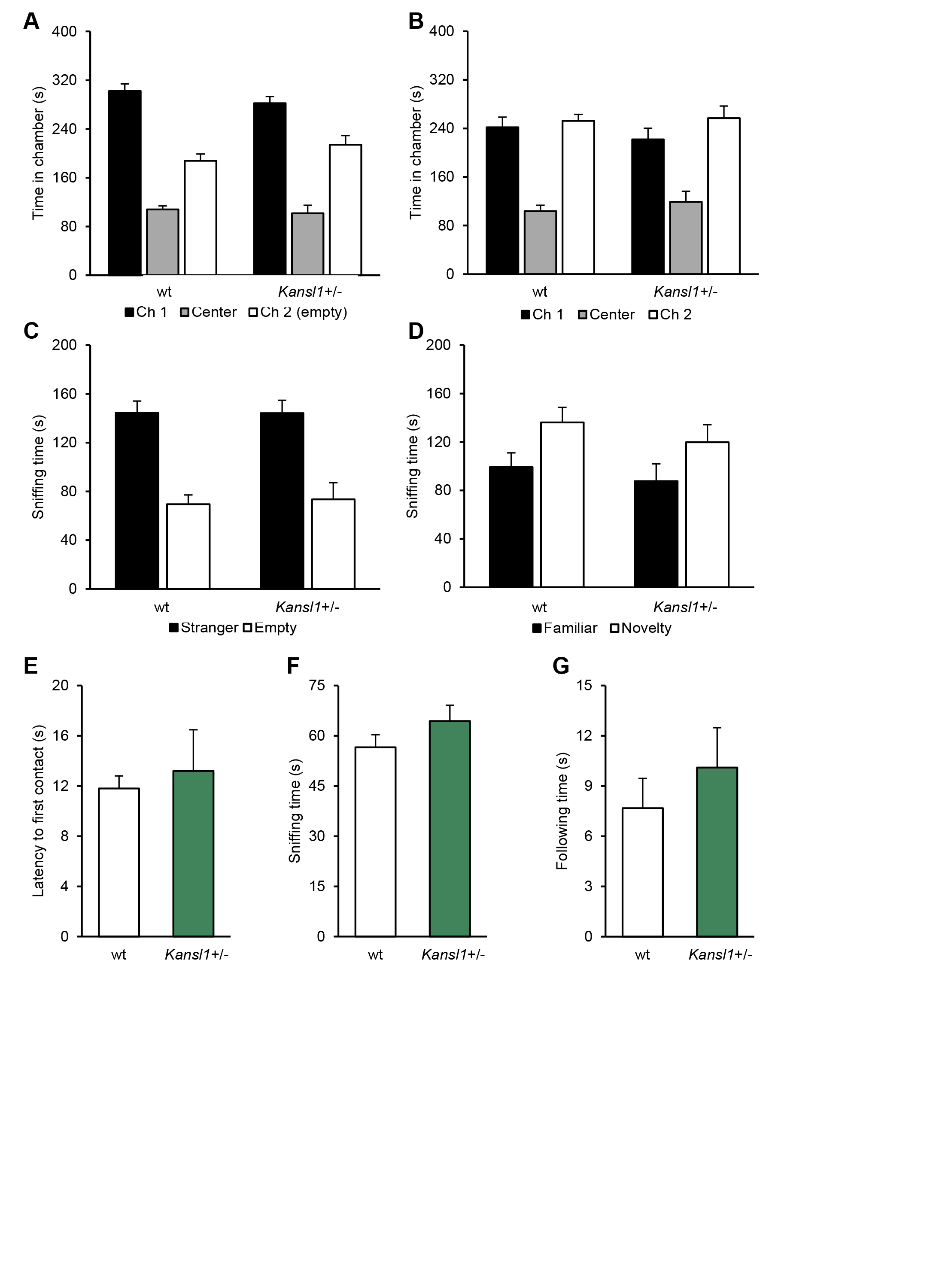

Supplement: S8 Fig — (A-D) Three-chamber test. (A, C) Social interest session. (B, D) Social discrimination session. (A-B) Time (s) in chambers during the social interest session (A) in which restrictive area of chamber 1 contains an unfamiliar mouse and during the social discrimination session (B) where chamber 1 and 2 restrictive areas contain respectively familiar and unfamiliar mice. (C-D) Corresponding time (s) spent to explore restrictive areas. Compared to wt littermates, Del/+ animals spent more time to interact with the stranger mouse during the social interest session. All genotypes show similar social discrimination capacities. (E-G) Social interaction test. Latency to first contact (E), sniffing time (F) and following time (G) (s) of 2 animals of similar genotype from different house cages putting together in an open-field during 10 min. The conclusion of the social behavior tests is that Kansl1+/- animals do not show social alterations. All graphs depict mean + s.e.m.. (TIF) [file pgen.1006886.s009.tif]
