# Supplementary material for: Mouse models of 17q21.31 microdeletion and microduplication syndromes highlight the importance of Kansl1 for cognition
Source: PLoS Genet. 2017 Jul 13;13(7):e1006886. doi: 10.1371/journal.pgen.1006886 (PMC5531616; doi:10.1371/journal.pgen.1006886)
Supplement: S1 Table — In the circadian activity test, both Del/+ and Dup/+ animals showed global vertical hypoactivity. Del/+ mice showed locomotor hypoactivity during dark and light phases and rearing hypoactivity during the light phase. Dup/+ mice showed rearing hypoactivity during habituation phase. No alteration of feeding behavior was noticed during the test. During open field sessions, Del/+ and Dup/+ mice showed respectively trends rearing hyperactivity and hypoactivity. No phenotype was observed in the elevated plus maze test. No difference in activity and anxiety was observed between Del/Dup mice and wt littermates. Data are mean ± SEM. (DOCX) [file pgen.1006886.s010.docx]

**Supplementary Table 1. Activity and anxiety characterization of the *Del-Dup* cohort.**

|  |  |  |  |  |  |
| --- | --- | --- | --- | --- | --- |
| **Test** | **Parameter** | **Genotype** | | | |
|  |  | ***Del/+*** | **wt** | ***Del/Dup*** | ***Dup/+*** |
| Circadian Activity | Hab ambulatory activity (count) | 223 ± 28 | 241± 17 | 230 ± 35 | 191 ± 23 |
|  | Hab vertical activity (count) | 326 ± 41 | 337 ± 58 | 324 ± 64 | 125 ± 21 |
|  | Dark ambulatory activity (count) | 316 ± 26** | 546 ± 37 | 535 ± 58 | 470 ± 58 |
|  | Dark vertical activity (count) | 492 ± 56 | 734 ± 84 | 686 ± 90 | 443 ± 50 |
|  | Light ambulatory activity (count) | 92.4 ± 12** | 172 ± 16 | 152 ± 20 | 155 ± 21 |
|  | Light vertical activity (count) | 70.6 ± 8.6** | 172 ± 27 | 173 ± 29 | 122 ± 23 |
|  | Total ambulatory activity (count) | 639 ± 56*** | 974 ± 48 | 932 ± 104 | 836 ± 94 |
|  | Total vertical activity (count) | 895 ± 78 | 1248 ± 145 | 1190 ± 165 | 699 ± 76* |
|  | Total food consumption (g) | 5.6 ± 0.3 | 5.7 ± 0.3 | 5.6 ± 0.2 | 5.3 ± 0.4 |
|  | Total water consumption (ml) | 5.6 ± 0.2 | 5.4 ± 0.3 | 5.6 ± 0.4 | 5.3 ± 0.4 |
| Open Field | Distance travelled (m) | 104 ± 4 | 105 ± 4 | 105 ± 5 | 95.4 ± 4.3 |
|  | Vertical activity (count) | 214 ± 23 | 167 ± 12 | 165 ± 24 | 120 ± 11 |
|  | Time in center (%) | 7.0 ± 0.7 | 8.7 ± 1.4 | 8.4 ± 1.2 | 10.2 ± 1.2 |
| Elevated Plus Maze | Arm entries (count) | 11.8 ± 1.2 | 14.2 ± 0.9 | 15.2 ± 1.1 | 10.9 ± 1.2 |
|  | Open arm time (%) | 2.7 ± 0.8 | 5.7 ± 0.9 | 7.9 ± 1.7 | 4.7 ± 1.1 |
