# Supplementary material for: Mouse models of 17q21.31 microdeletion and microduplication syndromes highlight the importance of Kansl1 for cognition
Source: PLoS Genet. 2017 Jul 13;13(7):e1006886. doi: 10.1371/journal.pgen.1006886 (PMC5531616; doi:10.1371/journal.pgen.1006886)
Supplement: S2 Table — No phenotype was observed in the Y maze test. Object recognition memory of mice was assessed with a retention delay of 3 h. In the first session (S1) of test, no difference in object exploration was noticed. In the retention session (S2), Del/+ mice showed recognition memory deficits. In the Morris water maze, no difference of spatial learning and memory was observed between the different genotypes. Mutant mice and wild-type littermates travelled the same distance and needed the same time to find the platform from first day to sixth day (D6) of acquisition. In the probe test (PT) on day 7, when the platform is removed, all mice spent a similar percentage of time in the target quadrant. In the fear conditioning test, all genotypes displayed similar level of activity in the conditioning session before footshock. Post-choc freezing was decreased in Del/+ and Del/Dup mice without significance. In the 6-min contextual session, Dup/+ animals showed a global higher freezing without significance. In cue sessions, Del/+ and Dup/+ mice displayed respectively lower and higher level of freezing in comparison with wt littermates. In the three-chamber sociability test, Del/+ mice showed an increased level of social interaction with the first stranger in first session (S1). Del/+ mice also presented trends for higher level of social interaction with the first stranger and the second stranger in the second session (S2). No alteration of social preference was found between genotypes. In the social interaction test, Del/+ animals displayed trends for shorter first contact latency and higher level of social interaction whereas Dup/+ mice displayed trends for longer first contact latency and lower level of social interaction in comparison with wt littermates. No phenotype was observed in rotarod and grip tests. Del/Dup mice showed similar performances than wt littermates. Data are mean ± SEM. (DOCX) [file pgen.1006886.s011.docx]

**Supplementary Table 2. Behavioral characterization of the *Del-Dup* cohort.**

|  |  |  |  | |  | |  |
| --- | --- | --- | --- | --- | --- | --- | --- |
| **Test** | **Parameter** | **Genotype** | | | | | |
|  |  | ***Del/+*** | | **wt** | | ***Del/Dup*** | ***Dup/+*** |
| Y Maze | Arm entries (count) | 25.1 ± 2.1 | | 22.5 ± 2.6 | | 20.0 ± 2.1 | 15.8 ± 2.1 |
|  | Alternation (%) | 64.9 ± 5.9 | | 64.6 ± 3.7 | | 63.8 ± 4.9 | 63.8 ± 5.9 |
| New Object Recognition 3 hour delay | S1 First object exploration (s) | 9.0 ± 0.6 | | 9.3 ± 0.8 | | 8.1 ± 1.0 | 6.2 ± 0.9 |
|  | S2 Former object exploration (s) | 4.6 ± 0.6 | | 3.4 ± 0.7 | | 2.7 ± 0.5 | 2.3 ± 0.7 |
|  | S2 Novel object exploration (s) | 6.3 ± 1.2 | | 6.0 ± 1.2 | | 4.5 ± 0.7 | 3.4 ± 0.5 |
|  | Discrimination index (%) | 56.0 ± 1.6* | | 63.7 ± 1.9 | | 62.4 ± 2.8 | 63.1 ± 3.3 |
| Morris water maze | D6 distance to platform (m) | 5.5 ± 0.6 | | 5.3 ± 0.7 | | 6.1 ± 1.3 | 5.1 ± 0.7 |
|  | D6 latency to platform (s) | 33.7 ± 4.7 | | 25.6 ± 4.4 | | 28.0 ± 7.0 | 30.1 ± 7.0 |
|  | Probe - time in target quad. (%) | 32.3 ± 3.3 | | 37.8 ± 3.5 | | 33.8 ± 3.6 | 41.9 ± 6.0 |
| Fear Conditioning | Baseline freezing (%) | 11.4 ± 2.1 | | 15.5 ± 3.4 | | 12.7 ± 3.4 | 25.1 ± 6.9 |
|  | Post-choc freezing (%) | 19.9 ± 3.3 | | 30.7 ± 5.6 | | 15.6 ± 3.1 | 31.7 ± 2.8 |
|  | Contextual freezing (%) | 42.7 ± 5.5 | | 54.1 ± 5.0 | | 52.6 ± 5.9 | 67.9 ± 5.9 |
|  | First Cue freezing (%) | 60.9 ± 5.7 | | 64.2 ± 4.8 | | 60.5 ± 6.9 | 79.7 ± 4.1 |
|  | Second cue freezing (%) | 50.8 ± 5.1** | | 73.8 ± 4.7 | | 59.0 ± 7.1 | 87.0 ± 3.9* |
| Three-Chamber Sociability | S1 First stranger exploration (s) | 206 ± 10* | | 169 ± 6 | | 171 ± 15 | 179 ± 16 |
|  | S2 First stranger exploration (s) | 101 ± 8 | | 71.4 ± 6.3 | | 87.6 ± 4.0 | 83.1 ± 12.6 |
|  | S2 Second stranger exploration (s) | 167 ± 13 | | 133 ± 11 | | 133 ± 11 | 124 ± 14 |
|  | Preference index (%) | 61.4 ± 3.3 | | 64.5 ± 2.6 | | 59.3 ± 2.8 | 60.3 ± 5.3 |
| Social Interaction | First contact latency (s) | 13.1 ± 4.6 | | 16.0 ± 4.5 | | 25.0 ± 10.4 | 44.0 ± 14.9 |
|  | Sniffing time (s) | 91.4 ± 10.9 | | 70.1 ± 9.4 | | 68.8 ± 7.0 | 50.4 ± 5.1 |
|  | Following time (s) | 13.9 ± 2.8 | | 8.8 ± 2.3 | | 6.5 ± 2.2 | 9.9 ± 4.3 |
| Rotarod | D1 Time on the rod (s) | 196 ± 14 | | 156 ± 11 | | 151 ± 18 | 139 ± 15 |
|  | D2 Time on the rod (s) | 223 ± 15 | | 190 ± 10 | | 199 ± 19 | 157 ± 13 |
|  | D3 Time on the rod (s) | 207 ± 15 | | 186 ± 11 | | 187 ± 19 | 141 ± 13 |
| Grip Test | Grip stength (g/g body weight) | 8.0 ± 0.2 | | 7.8 ± 0.2 | | 8.2 ± 0.3 | 8.2 ± 0.4 |
