# Supplementary material for: Mouse models of 17q21.31 microdeletion and microduplication syndromes highlight the importance of Kansl1 for cognition
Source: PLoS Genet. 2017 Jul 13;13(7):e1006886. doi: 10.1371/journal.pgen.1006886 (PMC5531616; doi:10.1371/journal.pgen.1006886)
Supplement: S3 Table — (DOCX) [file pgen.1006886.s012.docx]

**Supplementary Table 3 comparison of the absolute volumes of 83 brain structures observed in mutant *Del/+* and *Dup/+* animals with wt littermates by magnetic resonance imaging**

| **Structure** | **F-statistic** | **Del/+** | **wt** | **Del/Dup** | **Dup/+** |
| --- | --- | --- | --- | --- | --- |
| Dorsolateral entorhinal cortex | 45,13 | 3.15±0.19 | 2.76±0.13 | 2.77±0.073 | 2.43±0.087 |
| Perirhinal cortex | 39,99 | 2.64±0.15 | 2.36±0.1 | 2.38±0.076 | 2.08±0.07 |
| medial septum | 34,28 | 1.25±0.06 | 1.13±0.026 | 1.13±0.041 | 1.03±0.045 |
| Caudomedial entorhinal cortex | 34,27 | 5.89±0.41 | 5.39±0.2 | 5.35±0.17 | 4.62±0.22 |
| olfactory tubercle | 32,63 | 3.05±0.12 | 2.96±0.098 | 2.95±0.095 | 2.6±0.079 |
| Lateral orbital cortex | 31,09 | 3.6±0.12 | 3.26±0.13 | 3.27±0.15 | 2.98±0.091 |
| Medial orbital cortex | 30,08 | 2.05±0.12 | 1.79±0.055 | 1.79±0.1 | 1.62±0.083 |
| Ventral intermediate entorhinal cortex | 29,4 | 1.19±0.081 | 1.11±0.03 | 1.11±0.036 | 0.96±0.05 |
| nucleus accumbens | 28,62 | 4.06±0.23 | 3.8±0.11 | 3.8±0.1 | 3.43±0.079 |
| hypothalamus | 28,23 | 10.94±0.48 | 10.34±0.24 | 10.35±0.2 | 9.5±0.34 |
| Dorsal intermediate entorhinal cortex | 27 | 2.05±0.11 | 1.94±0.045 | 1.92±0.05 | 1.73±0.079 |
| basal forebrain | 26,93 | 5.12±0.27 | 4.83±0.11 | 4.88±0.14 | 4.39±0.12 |
| medial lemniscus/medial longitudinal fasciculus | 26,46 | 2.88±0.097 | 2.77±0.082 | 2.79±0.077 | 2.51±0.099 |
| Cingulate cortex: area 32 | 25,13 | 2.97±0.17 | 2.6±0.085 | 2.63±0.15 | 2.41±0.091 |
| Frontal association cortex | 25,07 | 7.55±0.47 | 6.67±0.23 | 6.71±0.32 | 6.18±0.22 |
| corticospinal tract/pyramids | 24,83 | 1.95±0.055 | 1.9±0.053 | 1.89±0.072 | 1.7±0.062 |
| Ectorhinal cortex | 22,18 | 2.61±0.16 | 2.45±0.11 | 2.47±0.095 | 2.16±0.079 |
| Ventral orbital cortex | 21,03 | 1.57±0.07 | 1.47±0.049 | 1.46±0.072 | 1.33±0.046 |
| globus pallidus | 20,88 | 3.31±0.17 | 3.21±0.13 | 3.19±0.083 | 2.85±0.1 |
| Cingulate cortex: area 29c | 20,63 | 1.99±0.14 | 1.85±0.064 | 1.82±0.091 | 1.64±0.05 |
| Posteromedial cortical amygdaloid area | 20,34 | 1.36±0.091 | 1.27±0.025 | 1.28±0.078 | 1.11±0.058 |
| amygdala | 20 | 11.06±0.59 | 10.59±0.3 | 10.57±0.37 | 9.55±0.35 |
| Primary motor cortex | 19,77 | 7.67±0.36 | 7.21±0.19 | 7.15±0.25 | 6.7±0.19 |
| crus 2: ansiform lobule (lobule 7) | 19,23 | 3.89±0.29 | 3.85±0.13 | 3.88±0.19 | 3.25±0.2 |
| pons | 18,3 | 17.71±0.92 | 16.65±0.51 | 16.81±0.62 | 15.24±0.65 |
| Primary somatosensory cortex | 16,95 | 5.4±0.21 | 5.1±0.16 | 5.05±0.15 | 4.76±0.21 |
| Secondary motor cortex | 16,62 | 6.74±0.33 | 6.43±0.16 | 6.34±0.25 | 5.94±0.15 |
| Piriform cortex | 15,13 | 11.29±0.59 | 11.17±0.26 | 11.13±0.29 | 10.25±0.17 |
| olfactory bulbs | 14,9 | 24.3±1.66 | 27.27±0.56 | 26.76±0.91 | 25.35±0.9 |
| periaqueductal grey | 14,56 | 4.1±0.32 | 3.75±0.15 | 3.79±0.17 | 3.45±0.12 |
| midbrain | 14,01 | 13.99±1.02 | 13.11±0.48 | 13.16±0.52 | 11.85±0.62 |
| striatum | 12,73 | 20.41±1.04 | 19.29±0.63 | 19.41±0.5 | 18.24±0.63 |
| cerebral peduncle | 12,23 | 2.3±0.12 | 2.34±0.065 | 2.33±0.057 | 2.13±0.097 |
| Primary somatosensory cortex: upper lip region | 12,1 | 6.7±0.24 | 6.39±0.22 | 6.3±0.2 | 6.02±0.27 |
| trunk of arbor vita | 11,84 | 4.37±0.27 | 4.37±0.14 | 4.39±0.16 | 3.94±0.16 |
| Temporal association area | 11,79 | 2.72±0.19 | 2.65±0.12 | 2.66±0.12 | 2.36±0.1 |
| colliculus: inferior | 11,77 | 5.27±0.36 | 5.07±0.22 | 5.07±0.21 | 4.57±0.19 |
| Cingulate cortex: area 24b | 11,69 | 1.58±0.11 | 1.46±0.026 | 1.47±0.078 | 1.37±0.04 |
| paramedian lobule (lobule 7) | 11,66 | 3.71±0.34 | 3.89±0.19 | 3.8±0.23 | 3.26±0.19 |
| colliculus: superior | 11,34 | 8.64±0.66 | 8.31±0.26 | 8.27±0.32 | 7.53±0.25 |
| anterior commissure: pars anterior | 11,29 | 1.38±0.097 | 1.49±0.031 | 1.5±0.054 | 1.39±0.039 |
| lobule 8: pyramis | 10,7 | 1.54±0.12 | 1.54±0.11 | 1.53±0.095 | 1.31±0.067 |
| Dorsal nucleus of the endopiriform | 9,73 | 1.43±0.085 | 1.37±0.057 | 1.36±0.037 | 1.28±0.035 |
| copula: pyramis (lobule 8) | 8,85 | 2.17±0.17 | 2.3±0.12 | 2.21±0.11 | 1.99±0.1 |
| crus 1: ansiform lobule (lobule 6) | 8,84 | 3.87±0.32 | 4.18±0.12 | 4.1±0.24 | 3.68±0.2 |
| medulla | 8,8 | 28.45±1.08 | 28.23±0.72 | 28.09±1.15 | 26.23±0.97 |
| hippocampus | 8,73 | 21.38±1.24 | 20.72±0.59 | 20.71±0.67 | 18.99±1.39 |
| thalamus | 8,63 | 17.37±1.12 | 16.82±0.53 | 16.8±0.48 | 15.62±0.65 |
| bed nucleus of stria terminalis | 8,49 | 1.45±0.078 | 1.41±0.053 | 1.41±0.051 | 1.32±0.036 |
| simple lobule (lobule 6) | 8,07 | 4.45±0.25 | 4.61±0.16 | 4.55±0.29 | 4.11±0.2 |
| cerebellar peduncle: middle | 7,77 | 1.24±0.044 | 1.31±0.042 | 1.28±0.056 | 1.2±0.06 |
| Cingulate cortex: area 24a | 7,58 | 1.8±0.079 | 1.72±0.071 | 1.72±0.1 | 1.61±0.064 |
| fimbria | 7,05 | 3.75±0.13 | 3.59±0.15 | 3.57±0.13 | 3.36±0.26 |
| lobule 10: nodulus | 6,38 | 1.18±0.083 | 1.27±0.04 | 1.25±0.055 | 1.17±0.068 |
| Secondary somatosensory cortex | 6,35 | 6.19±0.34 | 5.95±0.28 | 5.85±0.22 | 5.58±0.3 |
| Secondary visual cortex: lateral area | 6,29 | 2.93±0.25 | 2.84±0.15 | 2.89±0.12 | 2.62±0.1 |
| lobule 9: uvula | 6,12 | 2.68±0.23 | 2.78±0.16 | 2.75±0.14 | 2.46±0.16 |
| Cingulate cortex: area 30 | 5,96 | 2.52±0.17 | 2.5±0.12 | 2.46±0.14 | 2.27±0.09 |
| pre-para subiculum | 5,85 | 2.13±0.14 | 2.12±0.13 | 2.09±0.095 | 1.92±0.12 |
| Secondary auditory cortex: ventral area | 5,7 | 1.74±0.11 | 1.72±0.074 | 1.67±0.077 | 1.57±0.096 |
| Insular region: not subdivided | 5,7 | 7.55±0.33 | 7.29±0.36 | 7.17±0.24 | 6.94±0.26 |
| Primary somatosensory cortex: hindlimb region | 5,58 | 2.37±0.15 | 2.27±0.1 | 2.27±0.12 | 2.14±0.055 |
| optic tract | 5,35 | 1.63±0.13 | 1.69±0.043 | 1.7±0.059 | 1.57±0.076 |
| lobules 1-2: lingula and central lobule (ventral) | 5,32 | 1.43±0.13 | 1.59±0.083 | 1.61±0.11 | 1.52±0.083 |
| dentate gyrus of hippocampus | 4,82 | 3.8±0.33 | 3.65±0.13 | 3.66±0.15 | 3.41±0.23 |
| internal capsule | 4,77 | 2.86±0.16 | 2.9±0.11 | 2.89±0.081 | 2.72±0.1 |
| lateral septum | 4,66 | 3.36±0.21 | 3.28±0.11 | 3.23±0.11 | 3.09±0.16 |
| lobule 6: declive | 4,66 | 2.58±0.21 | 2.61±0.19 | 2.58±0.17 | 2.33±0.074 |
| Secondary auditory cortex: dorsal area | 4,65 | 1.6±0.11 | 1.55±0.07 | 1.56±0.06 | 1.46±0.07 |
| paraflocculus (PFL) | 4,61 | 3.41±0.13 | 3.66±0.23 | 3.7±0.19 | 3.48±0.19 |
| Secondary visual cortex: mediomedial area | 4,35 | 1.56±0.14 | 1.53±0.098 | 1.54±0.1 | 1.4±0.052 |
| Primary visual cortex: binocular area | 4,15 | 1.87±0.15 | 1.82±0.12 | 1.85±0.079 | 1.7±0.075 |
| Primary auditory cortex | 4,04 | 1.56±0.11 | 1.53±0.072 | 1.51±0.066 | 1.42±0.091 |
| anterior lobule (lobules 4-5) | 3,98 | 1.55±0.13 | 1.61±0.081 | 1.61±0.094 | 1.48±0.082 |
| Primary visual cortex: monocular area | 3,9 | 1.61±0.15 | 1.55±0.097 | 1.57±0.098 | 1.44±0.052 |
| Primary somatosensory cortex: forelimb region | 3,84 | 4.06±0.23 | 3.93±0.16 | 3.88±0.18 | 3.76±0.11 |
| corpus callosum | 3,67 | 13.74±0.96 | 13.78±0.58 | 13.7±0.63 | 12.8±0.66 |
| Primary visual cortex | 3,48 | 2.02±0.2 | 2.01±0.11 | 2.05±0.11 | 1.86±0.076 |
| lobule 3: central lobule (dorsal) | 3,17 | 1.74±0.16 | 1.82±0.13 | 1.86±0.15 | 1.66±0.14 |
| Primary somatosensory cortex: barrel field | 3,02 | 10.3±0.59 | 10.03±0.41 | 9.97±0.37 | 9.66±0.32 |
| lobules 4-5: culmen (ventral and dorsal) | 2,97 | 3.83±0.36 | 3.86±0.23 | 3.81±0.29 | 3.51±0.22 |
| lateral ventricle | 2,25 | 3.92±0.21 | 4.02±0.19 | 3.94±0.22 | 4.3±0.61 |
| third ventricle | 0,94 | 1.16±0.073 | 1.14±0.045 | 1.15±0.048 | 1.12±0.053 |
