# Supplementary material for: Mouse models of 17q21.31 microdeletion and microduplication syndromes highlight the importance of Kansl1 for cognition
Source: PLoS Genet. 2017 Jul 13;13(7):e1006886. doi: 10.1371/journal.pgen.1006886 (PMC5531616; doi:10.1371/journal.pgen.1006886)
Supplement: S4 Table — In the circadian activity test, Kansl1+/- mice showed locomotion hypoactivity in dark and light phases. No alteration of feeding behavior was noticed during the test. During open field sessions, Kansl1+/- animals showed rearing hyperactivity. Observation of repetitive behaviors in odorless home-cages revealed decreased level of digging and increased level of rearing in Kansl1+/- mice in comparison with wt littermates. Object recognition memory of mice was assessed with a retention delay of 3 h. In the first session (S1) of test, no difference in object exploration was noticed. In the retention session (S2), Kansl1+/- mice showed potent recognition memory deficits. In the fear conditioning test, no difference of activity in the conditioning session before footshock was observed between Kansl1+/- mice and wt littermates. In the 6-min contextual session, Kansl1+/- mice showed a reduced level of freezing. In cue sessions, Kansl1+/- mice also showed reduced level of freezing with significant statistical difference during the second cue. During the rotarod test, Kansl1+/- mice displayed an important improvement of motor coordination from the first day (D1) to the third day (D3) of test. No phenotype was observed in the grip test. Data are mean ± SEM. (DOCX) [file pgen.1006886.s013.docx]

**Supplementary Table 4. Behavioral characterization of *Kansl1*^+/-^ cohorts.**

|  |  |  |  | |
| --- | --- | --- | --- | --- |
| **Test** | **Parameter** | **Genotype** | | |
|  |  | ***wt*** | | ***Kansl1^+/-^*** |
| Circadian Activity | Hab ambulatory activity (count) | 253 ± 21 | | 243± 33 |
|  | Hab vertical activity (count) | 235 ± 47 | | 426 ± 89 |
|  | Dark ambulatory activity (count) | 522 ± 57 | | 314 ± 36 * |
|  | Dark vertical activity (count) | 872 ± 146 | | 1536 ± 427 |
|  | Light ambulatory activity (count) | 125 ± 8 | | 86.3 ± 10.3 ** |
|  | Light vertical activity (count) | 108 ± 15 | | 108 ± 18 |
|  | Total ambulatory activity (count) | 930 ± 77 | | 658 ± 71 * |
|  | Total vertical activity (count) | 1253 ± 194 | | 2126 ± 522 |
|  | Total food consumption (g) | 5.3 ± 0.2 | | 5.0 ± 0.5 |
|  | Total water consumption (ml) | 5.0 ± 0.2 | | 5.4 ± 0.3 |
| Open Field | Distance travelled (m) | 99.1 ± 7.1 | | 108 ± 5 |
|  | Rears (count) | 170 ± 21 | | 240 ± 24 * |
|  | Time in center (%) | 7.1 ± 1.2 | | 10.0 ± 1.3 |
| Repetitive Behaviors | Rearing (count) | 39.4 ± 2.8 | | 57.4 ± 6.3 * |
|  | Jumping (count) | 0.22 ± 0.22 | | 3.0 ± 2.3 |
|  | Climbing (count) | 10.4 ± 3.0 | | 14.8 ± 2.9 |
|  | Digging (count) | 13.1 ± 2.2 | | 3.9 ± 1.4 ** |
| Elevated Plus Maze | Arm entries (count) | 14.3 ± 1.8 | | 14.5± 2.2 |
|  | Open arm time (%) | 4.0 ± 2.3 | | 1.3 ± 0.6 |
| New Object Recognition 3 hour delay | S1 First object exploration (s) | 7.2 ± 0.5 | | 5.4 ± 0.7 |
|  | S2 Former object exploration (s) | 2.2 ± 0.4 | | 2.5 ± 0.5 |
|  | S2 Novel object exploration (s) | 4.3 ± 0.6 | | 2.8 ± 0.5 |
|  | Discrimination index (%) | 67.0 ± 2.6 | | 53.0 ± 1.2 *** |
| Fear Conditioning | Baseline freezing (%) | 9.9 ± 1.7 | | 13.7 ± 4.8 |
|  | Post-choc freezing (%) | 25.7 ± 5.7 | | 14.2 ± 3.3 |
|  | Contextual freezing (%) | 49.8 ± 7.1 | | 24.8 ± 4.7 * |
|  | First Cue freezing (%) | 61.8 ± 6.8 | | 42.5 ± 8.9 |
|  | Second cue freezing (%) | 69.8 ± 6.8 | | 32.7 ± 5.6 *** |
| Rotarod | D1 Time on the rod (s) | 64.2 ± 3.3 | | 89.3 ± 8.1 ** |
|  | D2 Time on the rod (s) | 77.7 ± 4.5 | | 164 ± 9 *** |
|  | D3 Time on the rod (s) | 114 ± 7 | | 202 ± 13 *** |
| Grip Test | Grip stength (g/g body weight) | 9.7 ± 0.4 | | 10.0 ± 0.3 |
